# Biotic and environmental dynamics through the Late Jurassic–Early Cretaceous transition: evidence for protracted faunal and ecological turnover

**DOI:** 10.1111/brv.12255

**Published:** 2016-02-17

**Authors:** Jonathan P. Tennant, Philip D. Mannion, Paul Upchurch, Mark D. Sutton, Gregory D. Price

**Affiliations:** ^1^ Department of Earth Science and Engineering Imperial College London South Kensington London SW7 2AZ U.K.; ^2^ Department of Earth Sciences University College London London WC1E 6BT U.K.; ^3^ School of Geography, Earth and Environmental Sciences Plymouth University Plymouth PL4 8AA U.K.

**Keywords:** biodiversity, biogeography, dinosaurs, extinction, faunal turnover, Gondwana, invertebrates, Laurasia, mass extinction, Mesozoic, micro‐organisms, radiation, selectivity, vertebrates

## Abstract

The Late Jurassic to Early Cretaceous interval represents a time of environmental upheaval and cataclysmic events, combined with disruptions to terrestrial and marine ecosystems. Historically, the Jurassic/Cretaceous (J/K) boundary was classified as one of eight mass extinctions. However, more recent research has largely overturned this view, revealing a much more complex pattern of biotic and abiotic dynamics than has previously been appreciated. Here, we present a synthesis of our current knowledge of Late Jurassic–Early Cretaceous events, focusing particularly on events closest to the J/K boundary. We find evidence for a combination of short‐term catastrophic events, large‐scale tectonic processes and environmental perturbations, and major clade interactions that led to a seemingly dramatic faunal and ecological turnover in both the marine and terrestrial realms. This is coupled with a great reduction in global biodiversity which might in part be explained by poor sampling. Very few groups appear to have been entirely resilient to this J/K boundary ‘event’, which hints at a ‘cascade model’ of ecosystem changes driving faunal dynamics. Within terrestrial ecosystems, larger, more‐specialised organisms, such as saurischian dinosaurs, appear to have suffered the most. Medium‐sized tetanuran theropods declined, and were replaced by larger‐bodied groups, and basal eusauropods were replaced by neosauropod faunas. The ascent of paravian theropods is emphasised by escalated competition with contemporary pterosaur groups, culminating in the explosive radiation of birds, although the timing of this is obfuscated by biases in sampling. Smaller, more ecologically diverse terrestrial non‐archosaurs, such as lissamphibians and mammaliaforms, were comparatively resilient to extinctions, instead documenting the origination of many extant groups around the J/K boundary. In the marine realm, extinctions were focused on low‐latitude, shallow marine shelf‐dwelling faunas, corresponding to a significant eustatic sea‐level fall in the latest Jurassic. More mobile and ecologically plastic marine groups, such as ichthyosaurs, survived the boundary relatively unscathed. High rates of extinction and turnover in other macropredaceous marine groups, including plesiosaurs, are accompanied by the origin of most major lineages of extant sharks. Groups which occupied both marine and terrestrial ecosystems, including crocodylomorphs, document a selective extinction in shallow marine forms, whereas turtles appear to have diversified. These patterns suggest that different extinction selectivity and ecological processes were operating between marine and terrestrial ecosystems, which were ultimately important in determining the fates of many key groups, as well as the origins of many major extant lineages. We identify a series of potential abiotic candidates for driving these patterns, including multiple bolide impacts, several episodes of flood basalt eruptions, dramatic climate change, and major disruptions to oceanic systems. The J/K transition therefore, although not a mass extinction, represents an important transitional period in the co‐evolutionary history of life on Earth.

## I. INTRODUCTION

The Late Jurassic–Early Cretaceous interval (164–100 Ma) represents a transitional period in the history of life on Earth, coeval with significant environmental fluctuations and changes in Earth systems processes (Hallam, 1986; Ogg & Lowrie, 1986; Weissert & Mohr, 1996; Hart *et al.*, 1997; Gröcke *et al.*, 2003; Weissert & Erba, 2004; Zorina *et al.*, 2008; Sager *et al.*, 2013). An emerging picture of this interval indicates that it was a time of elevated extinction in marine invertebrate faunas (Hallam, 1986; Alroy, 2010a), coinciding with a faunal turnover in low‐latitude, shallow marine faunas (Aberhan, Kiessling & Fursich, 2006; Klompmaker *et al.*, 2013). In vertebrate groups, there is similar evidence for a faunal turnover in the marine (Steel, 1973; Benson & Druckenmiller, 2014) and non‐marine (Upchurch *et al.*, 2011; Butler, Benson & Barrett, 2013; Nicholson *et al.*, 2015) realms, culminating in the apparent radiations of numerous major extant groups, including eusuchian crocodyliforms, marine turtles, and birds. Despite its importance, our understanding of this time interval is relatively poor compared to other Phanerozoic stratigraphic intervals. This is, in part, due to the lack of a robust, global chronostratigraphic framework for the Jurassic/Cretaceous (J/K) boundary (Zakharov, Bown & Rawson, 1996; Kudielka *et al.*, 2002; Sellwood & Valdes, 2006; Tremolada *et al.*, 2006; Wimbledon *et al.*, 2011; Guzhikov *et al.*, 2012; Li, Peng & Batten, 2013; Taylor *et al.*, 2014; Naipauer *et al.*, 2015). Other stratigraphic boundaries, such as the Cretaceous/Paleogene (K/Pg), have distinct geochemical markers that can be globally traced and dated, creating a near‐universally accepted definition of the boundary (e.g. Schulte *et al.*, 2010). However, no such discrete event is currently traceable for the J/K boundary, hindering correlations between austral, boreal, Tethyan, and non‐marine settings (Ogg & Lowrie, 1986; Bralower, Monech & Thierstein, 1989; Ogg *et al.*, 1991; Bornemann, Aschwer & Mutterlose, 2003; Žak *et al.*, 2011; Dzyuba, Izokh & Shurygin, 2013; Shurygin & Dzyuba, 2015). In addition, a general perceived lack of importance of the J/K boundary, compared to other well‐studied ‘event boundaries’, means that less research effort has been devoted to this time interval. This geological uncertainty has impacted upon our knowledge of the biological and evolutionary patterns and processes occurring through the J/K boundary. Currently, there is relatively little understanding of how biotic and abiotic patterns through this interval are linked, in spite of an emerging picture of biotic dynamics at this time (e.g. Alroy *et al.*, 2001; Upchurch *et al.*, 2011; Benson & Druckenmiller, 2014). By synthesising our current understanding of major Earth system dynamics and environmental changes, we aim to provide insight into the potential mechanisms that underpinned macroevolutionary changes through the J/K boundary.

### (1) Stratigraphic age of the J/K boundary

Recently, there has been substantial progress in determining the age of the J/K stratigraphic boundary, along with attempts at a global correlation (Wimbledon *et al.*, 2011). Mahoney *et al.* (2005) previously proposed an age of 145.5 ± 0.8Ma, a result that has since been widely accepted as the age of the J/K boundary (Ogg & Hinnov, 2012; although see below). Of particular note is the biostratigraphic use of calpionellids (calcareous microplankton), which have helped to refine the dating of the base of the Cretaceous (Blau & Grun, 1997; Hauser *et al.*, 2007; Casellato, 2010; Pruner *et al.*, 2010). Three biological markers, based on these calpionellids, have been identified as potential biozones marking the base of the Berriasian (and thus the J/K boundary), comprising: (*i*) the base of the *Calpionella* Zone and the sudden decline in species of *Crassicollaria*; (*ii*) the explosive radiation of small, globular forms of *Calpionella alpine* (supported by López‐Martínez, Barragán & Reháková, 2013); and (*iii*) the first appearances of two subspecies of *Nannoconus* (*N. steinmannii minor* and *N. kamptneri minor*; Wimbledon *et al.*, 2011). Furthermore, the base of magnetozone M18r has recently been identified as an indicator of the J/K boundary (Grabowski *et al.*, 2010, 2013). These primary markers are supported by a suite of secondary biostratigraphic and magnetostratigraphic indicators (Wimbledon *et al.*, 2011). However, Vennari *et al.* (2014) have argued for a younger, 140 Ma age for the J/K boundary, based on a combination of biostratigraphic markers, sedimentation rates, and isotopic analyses from an Argentinean site. As such, the absolute age of the J/K boundary remains uncertain, but a framework for its constraint is at least in place (Wimbledon *et al.*, 2011; Vennari *et al.*, 2014). Here, for the purposes of discussing the timing of events, we follow the absolute age proposed by Mahoney *et al.* (2005) of 145.5 Ma (i.e. the Tithonian–Berriasian boundary).

### (2) The J/K boundary as a possible extinction event

#### (a) *History of the J/K boundary extinction*

Early research into Phanerozoic macroevolutionary patterns led to the inclusion of the end‐Jurassic as one of eight mass extinction events based upon a 20% level of extinction (Raup & Sepkoski, 1982, 1984; Sepkoski, 1986; see online Appendix S1 for an extensive record of studies). Raup & Sepkoski (1982, 1984) noted that this extinction was geographically and taxonomically constrained, leading to debate about its identification as a mass extinction on the same order of magnitude as the ‘Big Five’ (Hoffman, 1985; Benton, 1986; Hallam, 1986; Raup & Boyajian, 1988; Hallam & Wignall, 1997). Numerous subsequent studies recovered evidence for a J/K extinction of similar magnitude to that of Raup & Sepkoski (1982) (Sepkoski, 1992, 1993; Rampino & Haggerty, 1995; Barnes *et al.*, 1996; Hallam, 1998; Benton, 2001; Ruban, 2005; Purdy, 2008). A recent analysis of Phanerozoic diversity by Melott & Bambach (2014) identified the J/K boundary as one of 19 major extinction intervals, measured as a proportion of extinction (19.9% generic extinction), based on the latest version of the Sepkoski compendium. Despite this relatively high apparent extinction intensity, a perceived lack of importance of the J/K transition means that it has been largely neglected and often only referred to in passing in studies of Phanerozoic biotic changes (e.g. Wignall, 2001; Bambach, 2006; Courtillot & Olson, 2007). This likely relates to the lack of unambiguous evidence for dramatic environmental shifts or catastrophic events, or identification of significant biotic fluctuations (i.e. the extinction of a major group) through this period, ultimately leading to the J/K boundary being downgraded from mass‐extinction status (Hallam, 1986; Hallam & Cohen, 1989; Hallam & Wignall, 1997; Bambach, Knoll & Sepkoski, 2002; Bambach, Knoll & Wang, 2004).

The magnitude, timing, and taxonomic inclusivity of a potential J/K extinction event has remained in a state of flux for different taxonomic groups (e.g. Raup & Sepkoski, 1982; Fara, 2000; Kiessling & Aberhan, 2007; Smith & McGowan, 2007; Benson & Butler, 2011; Alroy, 2014; see online Appendix S1). Direct comparisons between these and other studies are often confounded by the different metrics used to quantify changes in diversity (e.g. counts of originations, extinctions, or taxonomic occurrences), and the taxonomic level of study (i.e. species, genus or family‐level data) (e.g. Sepkoski, 1992, 1993; Alroy *et al.*, 2001; Bambach *et al.*, 2004; Foote, 2005). Furthermore, the treatment of taxonomic data has often varied greatly, with some studies taking a literal reading of the fossil record (e.g. Dodson, 1990; Peters, 2008; Rogov, Zakharov & Nikitenko, 2010), whereas others have applied a variety of sampling standardisation methods, ranging from the incorporation of information from phylogenetic relationships to create ‘ghost lineages’ (e.g. Young *et al.*, 2010; Fischer *et al.*, 2012; Mannion *et al.*, 2013), to the application of a range of subsampling protocols and modelling techniques (e.g. Barrett, McGowan & Page, 2009; Alroy, 2010a, 2010b; Upchurch *et al.*, 2011; Lloyd, Young & Smith, 2012). As such, methodological differences are likely to be at least partly responsible for disagreement in terms of whether the J/K boundary marks a mass extinction (e.g. Raup & Sepkoski, 1982, 1984), a period of inflated extinction (e.g. Lloyd *et al.*, 2008; Benson & Butler, 2011), or represents a time of normal rates of background extinction and faunal turnover (e.g. Benson & Druckenmiller, 2014).

#### (b) *Renewed evidence for an extinction event?*

Using large taxonomic occurrence databases and a range of sampling standardisation techniques, several recent studies (particularly of tetrapods) have noted a sharp decline in diversity around the J/K boundary (Smith & McGowan, 2007; Barrett *et al.*, 2009; Benson *et al.*, 2010; Benson & Butler, 2011; Mannion *et al.*, 2011, 2015; Upchurch *et al.*, 2011; Friedman & Sallan, 2012; Upchurch & Mannion, 2012). Much of this research has incorporated our increasing awareness and understanding of the links between the geological and fossil records, and the impact that heterogeneous sampling might have on obscuring our reading of palaeobiodiversity patterns. These more recent studies are starting to elucidate biotic dynamics through the J/K boundary in detail, revealing that there may be a hitherto undetected complexity. For example, in dinosaurs, the magnitude of extinction varies depending on the proxies used to model sampling effort or the geological record in estimating ‘residual’ diversity (Upchurch *et al.*, 2011). Benson *et al.* (2010) and Benson & Butler (2011) revealed a diversity decline in all marine tetrapod groups through the J/K boundary. Combined with the results of Upchurch *et al.* (2011), this indicates that potentially significant events were impacting upon both marine and terrestrial ecosystems.

Consequently, there are currently differing interpretations of the intensity, timing, geographical extent, and taxonomic inclusivity of any putative extinction event across the J/K boundary. Here, we present a review of our current understanding of diversity and macroevolutionary patterns through the Late Jurassic to Early Cretaceous interval, and place this in an environmental framework describing the major contemporaneous perturbations to Earth systems.

## II. ENVIRONMENTAL CHANGES DURING THE LATE JURASSIC–EARLY CRETACEOUS

### (1) Palaeogeography and palaeoceanography

**Figure 1:**
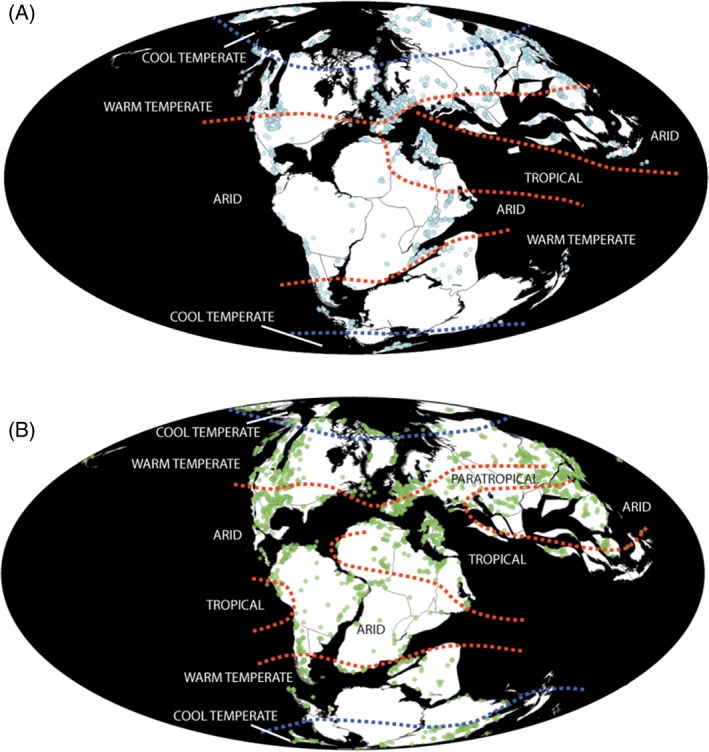
(A) Late Jurassic (Oxfordian–Tithonian) and (B) Early Cretaceous (Berriasian–Albian) global fossil occurrences, with climatic regions overlain. Fossil data extracted from *The Paleobiology Database*, November 2014 (http://paleobiodb.org/). Palaeoclimate data from Paleomap project (http://www.scotese.com/).

The continued fragmentation of Pangaea throughout the Late Jurassic and Early Cretaceous led to large‐scale tectonic processes, on both regional (e.g. Nürnberg & Müller, 1991; Monger *et al.*, 1994; Adatte *et al.*, 1996; Hathway, 2000; DeCelles, 2004) and global (e.g. Scotese, Gahagan & Larson, 1988; Scotese, 1991) scales, with accompanying palaeoceanographic changes including the initiation of the opening of the Central Atlantic (see Fig. 1). Salinity might have been slightly higher within the Late Jurassic proto‐Atlantic, particularly in restricted marginal basins (e.g. Sanford *et al.*, 2013) and at lower latitudes. Typically, however, the Atlantic must have been dominated by normal salinities as evidenced by the presence of fully marine faunas (e.g. Leinfelder, 1993), although it is possible that high sea levels and the configurations of the continents allowed high‐salinity waters in lower latitude epicontinental shelf seas to sink and form deep‐water masses (i.e. the warm saline bottom water model of Brass, Southam & Peterson, 1982). The opening of the South Atlantic during the Early Cretaceous rifting phases led to a gradual reduction in salinity (e.g. Evans, 1977). This rifting resulted in the connection of the present‐day Gulf of Mexico to southern Europe and the Tethys Ocean, with the Caribbean Ocean opening through continued motion of North and South America (Pindell & Kennan, 2009). In Africa, multiple rift phases were initiated during the latest Jurassic (Ford & Golonka, 2003), and Madagascar became isolated from Africa after the J/K boundary (Seton *et al.*, 2012). Sites with rocks spanning the J/K boundary have been targeted by the International Ocean Discovery Program (IODP) (and earlier incarnations). This program has included sites in (*i*) the Indian Ocean (Brown, 1992; Gradstein *et al.*, 1992; Kaminski, Gradstein & Geroch, 1992), with evidence for a cooler water regime; (*ii*) the Pacific Ocean, which is thought to have had a stable circulatory regime (Matsuoka, 1992; Ogg, Karl & Behl, 1992); and (*iii*) the Atlantic Ocean with a distinct North–South salinity gradient (Deroo, Herbin & Roucaché, 1983; Kotova, 1983; Gradstein *et al.*, 1992). In the Late Jurassic, western Tethys and Atlantic ecosystems were fuelled by a high‐nutrient flux, leading to high levels of phytoplankton and radiolarites, possibly driven by shifting circulatory regimes as continental configurations changed (Baumgartner, 1987; Weissert & Mohr, 1996; Danelian & Johnson, 2001).

Late Jurassic (Oxfordian) carbonate platforms experienced severe growth crises (Weissert & Mohr, 1996), and calcareous nannoplankton underwent a significant global radiation in the Tithonian–Berriasian (Weissert *et al.*, 1998; Bornemann *et al.*, 2003; Falkowski *et al.*, 2004; Weissert & Erba, 2004). However, the Early Cretaceous also saw a dramatic reduction in carbonate production, with a series of repeated ‘biocalcification crises’, notably in the Valanginian and Aptian (Weissert & Erba, 2004). ‘Disaster deposits’ from the Tethys Ocean resulted from localised but dramatic sea‐level falls and cooling episodes (Chatalov, Bonev & Ivanova, 2015).

### (2) Sea level and stratigraphy

**Figure 2:**
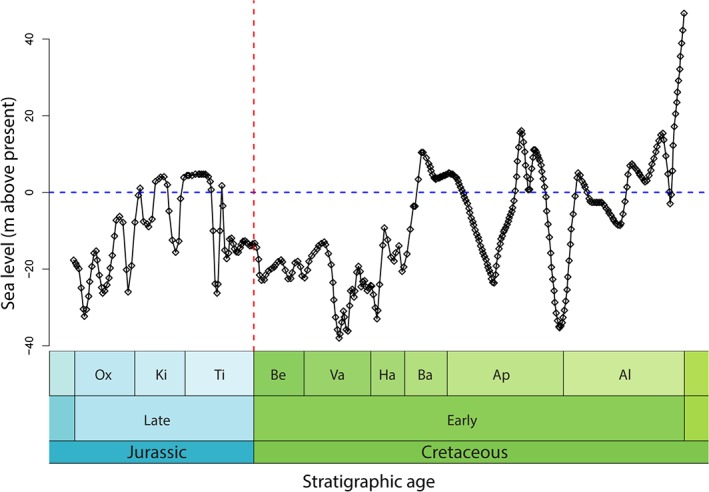
Sea‐level curve showing fluctuations through the Late Jurassic and Early Cretaceous. Data from Miller *et al.* (2005). Abbreviations: Ox, Oxfordian; Ki, Kimmeridgian; Ti, Tithonian; Be, Berriasian; Va, Valanginian; Ha, Hauterivian; Ba, Barremian; Ap, Aptian; Al, Albian.

Global (eustatic) sea‐level curves show a peak in the Kimmeridgian–early Tithonian, prior to a double‐dip decline and lowstand through the J/K boundary. This was followed by a slight rise to levels seen at the end of the Jurassic, before plummeting again in the Valanginian–Hauterivian (Haq, Hardenbol & Vail, 1987; Hallam, 1988, 2001; Miller *et al.*, 2005; Fig. 2) to the lowest sea level observed throughout the Cretaceous (Haq, 2014). Hallam (1986) proposed that this J/K boundary lowstand, and another during the Early Jurassic, were the principal drivers of major extinction events at the end‐Tithonian and end‐Pliensbachian. The proportion of evaporitic rocks in the Late Jurassic parallels this sea‐level pattern (Ronov *et al.*, 1980; Zorina *et al.*, 2008). Falling sea levels through the J/K transition decimated reef environments, as indicated by a marked decline in the areal extent and latest Jurassic diversity of reef‐building organisms (Kiessling, 2008; Foote, 2014). Black shales were widely deposited throughout the Late Jurassic and Early Cretaceous, often in marginal seas (Dypvik & Zakharov, 2012; Föllmi, 2012; Meyers, 2014). Multiple causes have been suggested for their deposition, including intense transgressive periods from rapidly changing sea levels (Lipinski, Warning & Brumsack, 2003), tectonic activity restricting flow patterns and increasing productivity (Wignall & Hallam, 1991; Weissert *et al.*, 1998), and decreased erosion rates and warmer, more arid climates (Kessels, Mutterlose & Ruffell, 2003; Föllmi, 2012). The Early Cretaceous saw several episodes of intense ocean water stagnation, possibly leading to anoxia, including the Valanginian Weissert and the late Hauterivian Faraoni oceanic anoxic events (Erba, Bartolini & Larson, 2004; Hu, Wagreich & Yilmaz, 2012; Mattioli *et al.*, 2014). However, Kujau *et al.* (2012) proposed that the Valanginian Weissert carbon excursion was not part of a global oceanic anoxic event, and that episodes of anoxia were instead restricted to the Atlantic, Pacific, and Southern Ocean, possibly with enhanced terrestrial carbon storage acting as the primary driver for the isotope excursion.

The Late Jurassic to Early Cretaceous eustatic sea‐level curve shows a positive correlation with fluctuations in global continental flooding (Budyko, Ronov & Yanshin, 1987; Hay *et al.*, 2006) and terriginous sedimentation (Haq *et al.*, 1987; Sahagian *et al.*, 1996; Ruban, 2010; Grabowski *et al.*, 2013; Zakharov *et al.*, 2014). The growth and decay of polar ice (Haq, 2014) and sea‐floor spreading (Hallam, 1988) might have acted as mechanisms to account for shorter‐term, relatively rapid, and at times large‐amplitude, falls in sea level in the Early Cretaceous. Additionally, Hay *et al.* (2006) observed that the Late Jurassic records the highest mass of sediment deposition in the Mesozoic, followed by a sharp decline through the J/K boundary. This finding is reflected on a regional scale, with the European rock record documenting a decline in sedimentary outcrop area from the Late Jurassic to Early Cretaceous, driven by a second‐order transgressive phase (Smith & McGowan, 2005, 2007). However, the fine‐scale correlation of sea‐level curves to global‐scale sedimentation patterns through the J/K boundary is currently poorly understood (Hallam, 1986; Zorina *et al.*, 2008; Ruban, 2011). This is a result of aforementioned variations in regional tectonics and eustacy‐driven sedimentation rates, leading to diachronous unconformities through the J/K boundary (Ogg *et al.*, 1991; Schnyder *et al.*, 2012).

The ratio of strontium isotopes (^87^Sr/^86^Sr) is an indicator of the rate of erosion of continental crust relative to enrichment from hydrothermal sources, and therefore acts as a proxy for marine sedimentation rate: peaks in the strontium curve correspond to minimum levels of associated sedimentation, and *vice versa* (Tardy, N'Kounkou & Probst, 1989). The Phanerozoic global strontium isotope curve decreases from the Ordovician up until the Middle/Late Jurassic boundary and increases across the J/K boundary (Jones *et al.*, 1994; Veizer *et al.*, 1999; Jones & Jenkyns, 2001; McArthur, Howarth & Bailey, 2001). This is generally concordant with long‐term systematic decreases in accommodation space and sedimentation rates associated with sea‐level regression (Budyko *et al.*, 1987; Hallam & Cohen, 1989; Hay *et al.*, 2006), as well as decreasing levels of humidity and weathering rates that continued until the end‐Jurassic (e.g. Ruffell & Batten, 1990; Hallam, Grose & Ruffell, 1991). The decrease in erosion rates that occurred across the J/K boundary is also tracked by a dramatic decline in chlorine flux into the oceans (Hay *et al.*, 2006). Furthermore, the ratio of *δ* ^34^S increased consistently across the J/K boundary (representing decreasing biologically driven sulfate reduction), which might have created toxic oceanic conditions (Kampschulte & Strauss, 2004). Together, these geochemical proxies provide strong evidence for major shifts in sedimentation patterns in concert with global‐scale marine environment perturbations.

Many regional studies have demonstrated that *δ* ^13^C values decreased through the J/K boundary, indicative of reduced oceanic productivity via a diminished flux of organic matter in the oceans and increasingly oligotrophic conditions (Weissert & Channell, 1989; Adatte *et al.*, 1996; Weissert & Mohr, 1996; Prokoph, Shields & Veizer, 2008; Zakharov *et al.*, 2014). In the Panboreal realm, this negative trend is coincident with a high abundance of spores and prasinophytes (unicellular green algae), the latter of which might relate to an algal bloom driven by disturbances to marine ecosystems and/or shifts in oceanic productivity (Zakharov *et al.*, 2014). However, the global radiation of calcareous plankton in the Tithonian–Berriasian (Weissert *et al.*, 1998; Bornemann *et al.*, 2003; Falkowski *et al.*, 2004; Weissert & Erba, 2004) is not fully expressed within the *δ* ^13^C record. Within the Boreal–Tethyan region, a positive carbon isotope excursion has been identified (Dzyuba *et al.*, 2013). Such variation has led to the idea that carbon isotopes may be useful in adding to the characterisation of the J/K boundary (e.g. Michalík *et al.*, 2009; Dzyuba *et al.*, 2013). In the Tethys, a negative carbon isotope excursion across the J/K boundary has been suggested (Grabowski *et al.*, 2010), possibly driven by increased continental weathering and erosion, or oxidation of organic‐rich sediments exposed during localised sea‐level transgression. These spatiotemporal variations indicate a geographically controlled scenario for carbon and oxygen isotope fluctuations over the J/K boundary, corresponding to varying rates of organic matter burial and biological productivity.

### (3) Climate

During the Late Jurassic, periods of enhanced continental erosion and oceanic productivity, combined with increased sedimentation rates, were driven by a dominantly tropical or arid climate with frequent monsoons at low latitudes (Hallam *et al.*, 1993; Weissert & Mohr, 1996; Fig. 1A). Information on variation in Late Jurassic and Early Cretaceous marine palaeotemperatures has been derived from oxygen isotope data based on well‐preserved marine molluscs (bivalves and belemnites) and brachiopods, largely from the Tethys and Boreal oceans (e.g. Gröcke *et al.*, 2003; Dera *et al.*, 2011; Price & Passey, 2013; Zakharov *et al.*, 2014). Following the Oxfordian warming period (e.g. Weissert & Erba, 2004; Jenkyns *et al.*, 2012), there was an increase in global temperatures in the Kimmeridgian (Anderson *et al.*, 1999; Scotese, Baucot & McKerrow, 1999; Bergman, Lenton & Watson, 2004; Price & Passey, 2013; Meyers, 2014). Increasing atmospheric temperatures through the Late Jurassic are consistent with results from the GEOCARBSULF model, although at a lower resolution (Berner, 2006, 2009). Numerous studies indicate a cooling and aridity episode in the late Tithonian (a ‘cold snap’), followed by a temperature and humidity increase during the Berriasian (Hallam *et al.*, 1991; Gröcke *et al.*, 2003; Jenkyns *et al.*, 2012; Grabowski *et al.*, 2013; Zhang *et al.*, 2014). These ‘cold snaps’ also occurred at the Middle–Late Jurassic boundary and early Aptian (Jenkyns *et al.*, 2012), and have been associated with marine biotic crises (McAnena *et al.*, 2013). When aridity reached its peak development during the earliest Cretaceous, arid regions extended across much of southern Eurasia, whilst higher latitudes were more humid (Hallam *et al.*, 1991). Cooling might have been more significant at higher latitudes in the Boreal and Tethyan realms, creating a stronger latitudinal climatic gradient up to the J/K boundary (Žak *et al.*, 2011), although some evidence suggests that high northern latitudes experienced a coupled oceanic–atmospheric warming (Zakharov *et al.*, 2014). Following cooling during the Tithonian and Berriasian, gradual warming occurred through the Early Cretaceous, beginning in the Valanginian, and possibly punctuated by short, cooler interludes (Weissert & Channell, 1989; Berner, 2001; Bice, Huber & Norris, 2003; Price & Rogov, 2009; Hannisdal & Peters, 2011; Jenkyns *et al.*, 2012; Price & Passey, 2013). For example, Price & Mutterlose (2004) and Price & Passey (2013) suggested that temperatures during the late Valanginian were consistent with sub‐freezing polar conditions. The gradual warming trend is detected in numerous regional localities, conceivably related to an increase in volcanic activity throughout the Early Cretaceous creating a ‘greenhouse’ world (Wang *et al.*, 2006; Sager *et al.*, 2013; see Section (4). From the Valanginian onwards, this volcanism might also be associated with concurrent oceanic anoxic events (Erba, 2004; Erba *et al.*, 2004). However, the idea of Early Cretaceous cool interludes conflicts with the work of other authors; instead, these studies indicate that the Earth experienced consistently warm and stable temperatures, with a shallow latitudinal temperature gradient (Hay, 2008; Littler *et al.*, 2011; Pouech *et al.*, 2014). Furthermore, the Northern and Southern hemispheres might have experienced markedly different climatic regimes throughout the Late Jurassic and Early Cretaceous, which might have been a consequence of the relative positions of the major oceans and landmasses (Jenkyns *et al.*, 2012).

In terms of terrestrial temperature variation across the J/K boundary, data are much more limited. Data for the Late Cretaceous (e.g. Spicer & Parrish, 1990) are relatively abundant, and indicate peak temperatures in the mid‐Cretaceous, followed by a Late Cretaceous decline. As these data are largely consistent with the marine record, it might suggest that the patterns of marine temperature change through the J/K boundary are matched in the terrestrial realm. Indeed, Abbink *et al.* (2001) described similar changes on the basis of quantitative sporomorph data, whereby climate from the middle Oxfordian to the Berriasian was characterised by stepwise warming and increasing aridity, followed by slight cooling. However, new data from terrestrial records are required to determine whether marine temperatures are a good proxy for continental patterns during this interval.

### (4) Volcanism

Volcanic emissions have the potential to transmit large volumes of toxic and other harmful materials into the atmosphere, and in general can have the following climatic and environmental effects: (*i*) lowering air temperatures through direct insolation from ash and sulphate aerosols; (*ii*) increasing atmospheric toxicity and poisoning; (*iii*) acid rain and biocalcification crises; (*iv*) increasing atmospheric temperatures through release of greenhouse gases; and (*v*) ocean anoxia (e.g. Bluth *et al.*, 1993; Robock, 2000; Wignall, 2001; Schaller, Wright & Kent, 2011). The Shatsky Rise, a vast shield volcano with a surface area of around 480000 km^2^, formed in the northwest Pacific Ocean at the J/K boundary (Sager *et al.*, 2013). Recent ^40^Ar/^39^Ar age determinations of basaltic lava samples from Tamu Massif, the oldest and largest edifice of the submarine Shatsky Rise, provide an age of 144 Ma (Geldmacher *et al.*, 2014), coincident with the J/K boundary. The impact of this extensive volcanism on Earth system cycles is currently poorly understood; however, it was significant enough to have affected the palaeotectonic motion of adjacent plates (Seton *et al.*, 2012).

**Figure 3:**
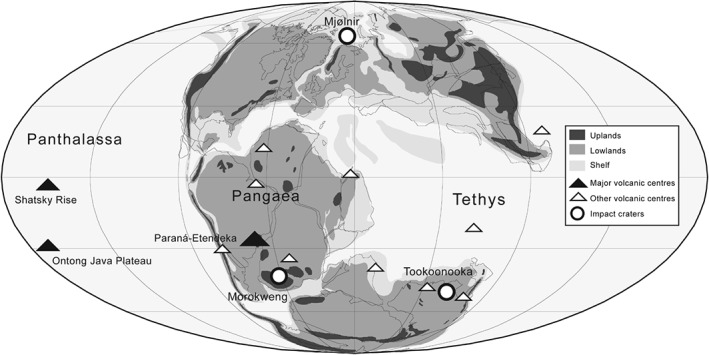
Late Jurassic and Early Cretaceous (Oxfordian–Albian) palaeogeographic map showing the locations of major flood basalts, minor volcanic activity, and bolide impacts. See text for details (Sections (4 and (5).

Accompanying the Shatsky volcanism, and coincident with ongoing Gondwanan fragmentation (Wignall, 2001; Segev, 2002), was a host of mantle plume‐related and smaller‐scale volcanic activity (Fig. 3). These include: (*i*) 10–20 km thick sequences in the Jurassic to Early Cretaceous of Chile (Vergara *et al.*, 1995); (*ii*) evidence for a plume event (Wilson & Guiraud, 1998) recorded in the Oxfordian deposits of northern Brazil (Baksi & Archibald, 1997), north‐east Africa (Segev, 2000), and Western Africa (Maluski *et al.*, 1995), the latter of which continued erupting into the Valanginian–Hauterivian; (*iii*) plume‐associated activity from the J/K boundary of the Liberian margin (Garfunkel, 1998) and the Equatorial Atlantic (southern India, northern South Africa, southeast Australia, the Antarctic peninsula, and Patagonia), concurrent with the final stage of the Karoo igneous province (e.g. Vaughan *et al.*, 1998; Féraud *et al.*, 1999); (*iv*) Berriasian–Hauterivian mantle plume activity in northern Israel (Segev, 2009); and (*v*) a 1500 km wide magmatic province initiated in the Hauterivian–Barremian of Australia (Bryan *et al.*, 1997). Alongside these are the extensive Paraná flood basalts of South America and the Etendeka Traps of Namibia that were jointly emplaced during a rifting phase throughout the late Valanginian and Hauterivian, possibly related to initiation of seafloor spreading in the South Atlantic (Harry & Sawyer, 1992; Jerram *et al.*, 1999; Seton *et al.*, 2012). The Paraná event (133 Ma) is estimated to have produced approximately 1.5 million km^3^ of volcanic rock, implying a rate consistent with a mantle plume origin (Renne *et al.*, 1992), and around three times the volume of the end‐Cretaceous Deccan volcanism. Although a link between the Paraná‐Etendeka igneous province and the Weissert Event has been suggested previously (Erba *et al.*, 2004), available dates suggest that this is unlikely (e.g. Martinez *et al.*, 2013), and that the environmental impacts of the volcanism were minimal due to its longevity (Dodd, Niocaill & Muxworthy, 2015). In the latest Barremian, the single largest volcanic province known on Earth was emplaced in the southwest Pacific, the Ontong Java Plateau (approximately 50 million km^3^; Coffin & Eldholm, 1994). This is concurrent with, and potentially a driver of, the marine biotic changes that culminated in oceanic anoxic event 1 (OAE1a) in the early Aptian (Bralower *et al.*, 1994; Wignall, 2001; Weissert & Erba, 2004). It is likely that this intensification of plume‐related activity is related to the increased continental fragmentation rates in the Late Jurassic to Early Cretaceous (see Section (1).

### (5) Bolide impacts

Impacts from extra‐terrestrial objects have the potential to cause massive disruption to Earth systems. These range from shockwaves, earthquakes, wildfires and tsunamis upon impact, to depletion of ozone and the release of carbon dioxide and sulphur into the atmosphere, causing an enhanced greenhouse effect and acid rain (e.g. O'Keefe & Ahrens, 1989; Toon *et al.*, 1997; Kaiho *et al.*, 2001). There are three known bolide impacts that are approximately contemporaneous with the J/K boundary (Fig. 3): (*i*) the 70–80 km diameter Morokweng impact crater in the Kalahari Desert, South Africa, dated at 145 ± 2 Ma (Corner *et al.*, 1997; Hart *et al.*, 1997; McDonald *et al.*, 2001; Henkel, Reimold & Koeberl, 2002; Reimold, Armstrong & Koeberl, 2002) and recognised from gravity and magnetic anomalies, as well as a bed enriched in extra‐terrestrial elements with evidence of external impact (note that new, preliminary data suggests that the original diameter of this structure might have been up to 240 km, 1.3–2 times the size of the end‐Cretaceous Chicxulub impact crater; Misra *et al.*, 2014); (*ii*) the 40 km wide Mjølnir crater in Norway, dated as 142 Ma (Dypvik, Gudlaugsson & Tsikalas, 1996); and (*iii*) the 22 km wide crater at Gosses Bluff, Northern Territory in Australia, dated at 142.5 Ma (Milton *et al.*, 1972; Milton & Sutter, 1987). Additionally, the 55 km wide Tookoonooka impact structure from Queensland, Australia, has been dated to the Early Cretaceous at 125 ± 1 Ma (Bron & Gostin, 2012). Alongside these larger impacts, there were at least nine additional smaller (1–20 km wide) impacts from the Late Jurassic to Early Cretaceous, based on The Earth Impact Database (http://www.passc.net/EarthImpactDatabase/), in South America, Europe, Africa, Australia, and Asia. Presently, geochemical data are limited in extent, but there are multiple regional anomalies whereby trace metals (e.g. iron, cobalt and nickel, and possibly iridium and chromium) are enriched around the J/K boundary, suggesting extra‐terrestrial input on a global scale (Zakharov, Lapukhov & Shenfil, 1993; Kudielka *et al.*, 2002; McDonald *et al.*, 2006; Mizera, Randa & Kostak, 2010). On a regional scale, dissipation of the energy release associated with the Mjølnir impact is estimated to have caused several short, near‐field perturbations, including large‐magnitude earthquakes, displacement of a considerable amount of material from the impact site, and debris flows and high‐amplitude tsunami waves (Dypvik *et al.*, 2006). Combined with evidence of large‐scale volcanism, the impact record provides strong evidence for multiple catastrophic events around the J/K boundary.

## III. BIOTIC CHANGES DURING THE LATE JURASSIC–EARLY CRETACEOUS TRANSITION

### (1) Incomplete and biased sampling in the fossil record

Early investigations into the trajectory of diversity on a geological time scale typically used raw counts of fossil taxa, i.e. numbers of species, genera, and/or families through time (e.g. Sepkoski, 1982, 1984, 1986). However, our uneven sampling of the fossil record means that a literal reading is likely to be problematic, with observed patterns in diversity potentially artefacts of a biased record. There are two main modes in which the fossil record can be biased by sampling: geological (including taphonomic biases) and anthropogenic. The former concerns the nature in which the geological record preserves the biological record, through the processes of burial and decay, and the amount of fossil‐bearing rock preserved in a particular time and place. This influences the frequency of opportunities to sample fossils, or the fossilisation potential of a particular depositional environment (e.g. Smith, Gale & Monks, 2001; McGowan & Smith, 2008; Smith & McGowan, 2008). Geological factors such as uplift and erosion also affect the accessibility of fossiliferous sediments. Anthropogenic sampling biases include the way we have sampled the fossil record, through increased collecting intensity at well‐known sites, and/or time intervals of special interest (e.g. Upchurch *et al.*, 2011), but also include other human factors such as economics, political situations, or legal concerns.

Before we can start to explore and interpret macroevolutionary patterns in the fossil record, we need to be able to understand and deal with such sampling biases. Fortunately, there are methods for ameliorating these biases. Modelling techniques can be used that estimate the portion of standing diversity that cannot be explained by our sampling of the geological and fossil records, using sampling proxies (e.g. numbers of collections, fossil‐bearing stratigraphic formations, rock outcrop area). These methods seek to explain whether diversity is driven by: (*i*) sampling bias; (*ii*) an external ‘common cause’, such as sea level; and/or (*iii*) redundancy, resulting from the non‐independence of sampling metrics and diversity (Benton *et al.*, 2011). Resulting ‘residual’ diversity curves from these modelling approaches represent biological deviations from a null model in which observed diversity is driven purely by sampling (Smith & McGowan, 2007; Barrett *et al.*, 2009; Lloyd, 2012). However, the use and appropriateness of sampling proxies has been questioned by some studies (Crampton *et al.*, 2003; Benton *et al.*, 2013; Dunhill, Hannisdal & Benton, 2014). Additional modelling approaches include probabilistic estimation using capture–mark–recapture models developed from those used in ecology (e.g. Nichols & Pollock, 1983; Liow & Nichols, 2010; Liow, 2013).

Subsampling techniques such as rarefaction account for heterogeneous sample sizes, setting a baseline subsampling threshold to the poorest‐sampled bin as a measure of relative quality (Raup, 1975; Jackson & Johnson, 2001). Three new modes of randomised subsampling analysis were introduced by Alroy *et al.* (2001) based on subsampling from taxonomic occurrence lists using various weighting exponents. The shareholder quorum subsampling (SQS) technique was subsequently developed by Alroy (2010a), and assigns different weights to species occurrences depending on the frequency of their occurrence as a protocol for ‘fair’ subsampling.

The phylogenetic relationships between taxa provide an additional source of information that can be utilised to reconstruct past diversity. A time‐calibrated tree incorporates ‘ghost lineages’ that represent gaps in the fossil record, with the first appearance time of a taxon extended back to that of its oldest known sister taxon occurrence (e.g. Wagner, 2000; Lane, Janis & Sepkoski, 2005; Cavin & Forey, 2007). Construction of ‘ghost lineages’ can increase diversity in time bins where we know a taxon must have been present, but has not yet been sampled. However, there are drawbacks to this approach, primarily in that it cannot account for range extensions from the last appearance to the true extinction date of a lineage; i.e. ‘zombie’ lineages (Lane *et al.*, 2005), which can result in a Signor–Lipps effect of an artefactually smeared out extinction.

Recent analytical studies have built on and surpassed earlier research into long‐term macroevolutionary patterns *via* the development of databases such as *The Paleobiology Database* (http://www.paleobiodb.org) and *Fossilworks* (http://www.fossilworks.org), synchronous with the progress in analytical techniques outlined above. Macroevolutionary studies that include the J/K interval have explored the effects of uneven sampling on vertebrate diversity at regional and global levels (e.g. Upchurch *et al.*, 2011; Butler *et al.*, 2013; Lloyd & Friedman, 2013; Benson & Druckenmiller, 2014; Mannion *et al.*, 2015; Nicholson *et al.*, 2015), and are supplemented by a wealth of taxonomic and systematic work. Combined with work on the diversity dynamics of marine invertebrates (e.g. Alroy, 2010a, 2010b), these recent studies are beginning to reveal a much more nuanced view of macroevolutionary patterns across the J/K boundary.

### (2) The quality of the Late Jurassic–Early Cretaceous fossil record

Numerous studies have documented changes in sampling quality over the J/K boundary. Notable examples include the sharp decline in fossiliferous marine‐ (Benson *et al.*, 2010; Benson & Butler, 2011), pterosaur‐ (Butler *et al.*, 2009c), and dinosaur‐bearing collections and formations (Upchurch *et al.*, 2011) in the earliest Cretaceous. Continental (Benson & Butler, 2011) and marine (Smith, 2001) outcrop areas show a shallow and steep decline, respectively, over the J/K boundary, with implications for the availability of possible fossil‐bearing sites to sample. An additional measure of quality for the fossil record is specimen completeness, or the proportion of the skeleton that is known for a particular taxon as a whole. For example, sauropod dinosaurs (Mannion & Upchurch, 2010b), birds (Brocklehurst *et al.*, 2012), pterosaurs (Dean, Mannion & Butler, 2016) and ichthyosaurs (Cleary *et al.*, 2015) all show reductions in average skeletal completeness over the J/K boundary, coincident with a drop in diversity in the first three groups (see Section III).

**Figure 4:**
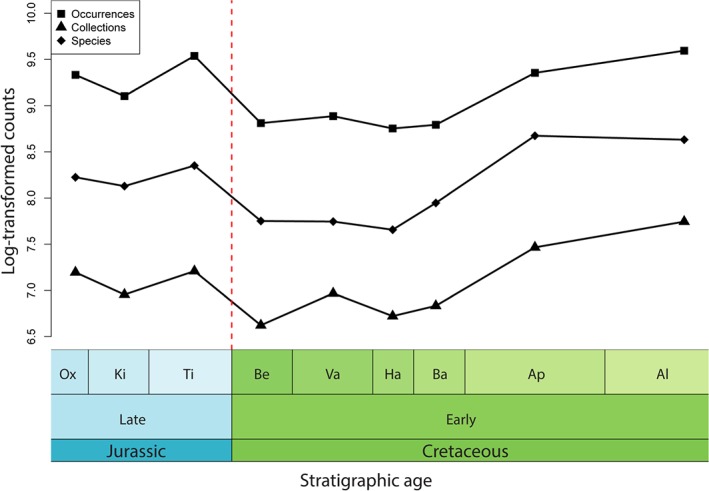
Global taxonomic diversity curve for all animal species, individual fossil collections and occurrences. The Jurassic/Cretaceous boundary is marked by a red line. Data extracted from *The Paleobiology Database*, 8 January, 2014 (http://paleobiodb.org/). Constructed using the *geoscale* package (Bell, 2014) in R (version 3.1.1; R Core Team, 2014). Abbreviations as in Fig. 2.

**Figure 5:**
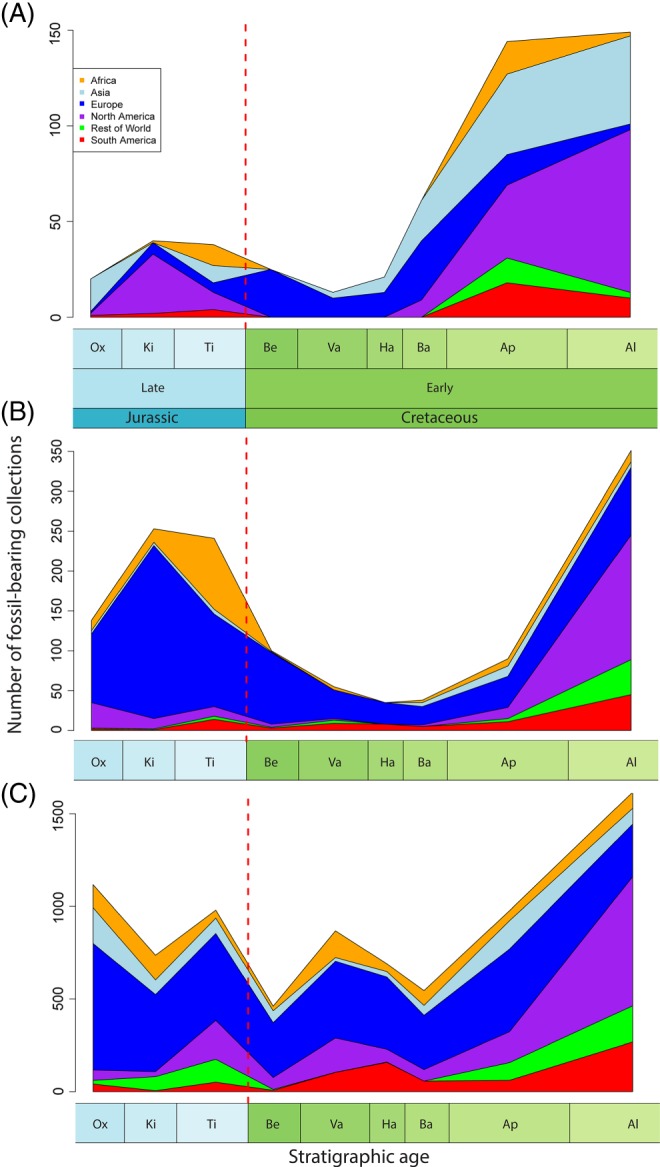
Late Jurassic and Early Cretaceous fossil‐bearing collections based on continental location. (A) Terrestrial collections (vertebrates only); (B) marine collections (vertebrates only; and (C) marine collections (invertebrates only). Data from *The Paleobiology Database*, accessed January, 2015 (http://paleobiodb.org/). Collections represent irreducible and discrete fossil‐bearing localities. Only those that could be dated to stage‐level resolution were included (S2). Constructed using the *geoscale* package (Bell, 2014) in R (version 3.1.1; R Core Team, 2014). Abbreviations as in Fig. 2.

Using data from *The Paleobiology Database* and *Fossilworks* (downloaded on 8 January, 2015), the total numbers of taxonomic occurrences, fossil‐bearing collections, and raw species diversity all show substantial declines over the J/K boundary (Fig. 4). The way in which we have sampled the fossil record spatially also affects our understanding of Late Jurassic and Early Cretaceous biotic patterns (e.g. Mannion *et al.*, 2012, 2015; Vilhena & Smith, 2013) (Fig. 5). The northern hemisphere is generally sampled better than the southern hemisphere in the Late Jurassic (Fig. 1A), with a shift to increased global sampling effort for much of the Early Cretaceous (Fig. 1B). For terrestrial vertebrates, the Late Jurassic record is dominated by North American and East African collections, with multiple fossil‐bearing sites from the well‐sampled Morrison and Tendaguru formations, respectively (Fig. 5A). Much of our understanding of latest Jurassic terrestrial diversity comes from Lagerstätten, such as the Solnhofen Limestone (Tithonian of southeastern Germany; Wellnhofer, 1970) and the Daohugou Biota (Oxfordian of northeastern China; Sullivan *et al.*, 2014). The earliest Cretaceous is devoid of terrestrial Lagerstätten, with the stratigraphically oldest deposits known from the Barremian of Spain (Las Hoyas), the Barremian–Aptian of China (Jehol), and the Aptian–Albian of Brazil (Crato and Santana). In the earliest Cretaceous (Berriasian–Hauterivian), terrestrial vertebrate fossils are known primarily from Asia and Europe, whereas North American and Gondwanan occurrences become common again only from the Barremian onwards. There is a decline in the numbers of terrestrial vertebrate collections over the J/K boundary, before a dramatic increase in the Barremian, with around three times the number of fossil‐bearing collections known in the Aptian–Albian than for any time during the Late Jurassic; however, this is likely to be largely driven by the Lagerstätten effect, as noted above. Australia and Antarctica completely lack a terrestrial fossil record through most of the Late Jurassic–Early Cretaceous, with the first occurrences known from the Aptian and Coniacian, respectively.

Almost our entire knowledge of Late Jurassic marine vertebrates comes from Europe, with a significant contribution from Africa (Fig. 5B). There is a dramatic decline in collecting effort through the J/K boundary, which begins to recover only in the Aptian, before North American collections dominate from the Albian onwards. A similar pattern is known for Late Jurassic marine invertebrates (Fig. 5C), with African collections contributing much to our knowledge of Kimmeridgian diversity, and North America to our understanding of Tithonian diversity. There is a substantial decline in the number of marine invertebrate collections over the J/K boundary in all geographic regions, but a rapid recovery in the Valanginian, when Gondwanan collections begin to contribute greatly. European collections dominate our knowledge of Early Cretaceous marine and terrestrial faunas (see also Smith & McGowan, 2007), exceeded only by North American collections in the Albian.

These patterns contribute to our understanding of the temporal and spatial biases that determine our knowledge of Mesozoic biota. Below, we provide a detailed review of biotic patterns during the Late Jurassic–Early Cretaceous transition.

### (3) Vertebrates

#### (a) *Dinosaurs*

**Figure 6:**
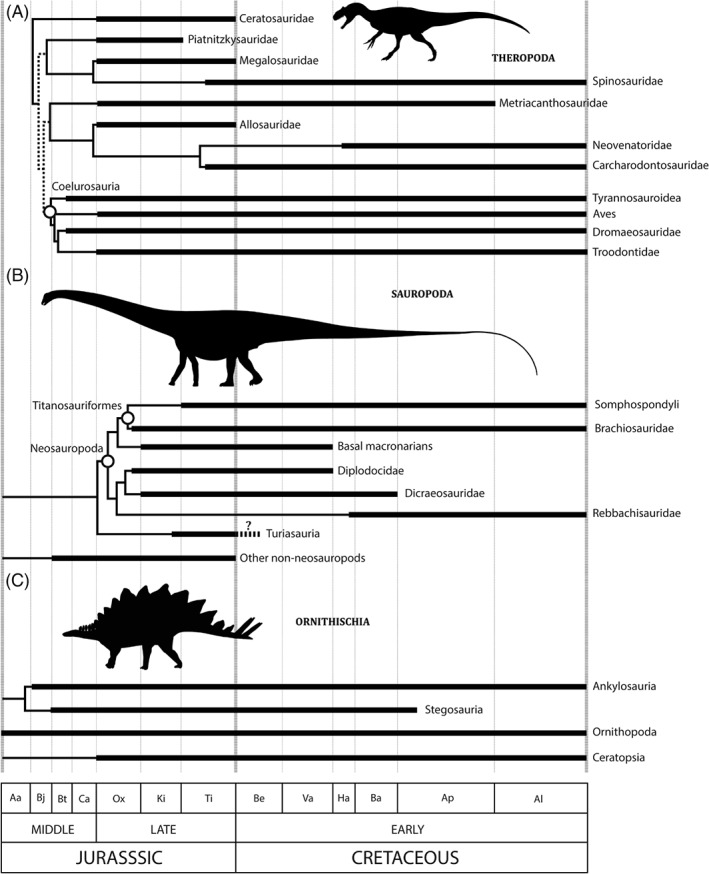
Stratigraphic ranges of major Jurassic–Cretaceous theropod (A), sauropod (B), and ornithischian (C) dinosaur clades through the Middle Jurassic to Early Cretaceous. Theropods are adapted from figure 22 of Carrano, Benson & Sampson (2012); sauropods are adapted from figure 22 of Mannion *et al.* (2013). Clade dates are based on those available from *The Paleobiology Database*, and supplemented from the primary literature. See text for details. Silhouettes from PhyloPic (http://phylopic.org/); *Allosaurus fragilis* and *Diplodocus* by Scott Hartman (CC BY‐SA 3.0), and *Stegosaurus* by Andrew Farke (CC BY 3.0). Abbreviations as in Fig. 2; additional abbreviations: Aa, Aalenian; Bj, Bajocian; Bt, Bathonian; and Ca, Callovian.

Of all Mesozoic vertebrate groups, dinosaurs have the best‐sampled and most‐studied fossil record. Non‐avian dinosaur diversity halved from the Tithonian to Berriasian (Lloyd *et al.*, 2008; Barrett *et al.*, 2009; Upchurch *et al.*, 2011). This pattern is geographically focused in taxa from Europe and North and South America, with Africa and Asia relatively unaffected (Upchurch *et al.*, 2011). However, the precise details and magnitude of this diversity reduction are obfuscated by relatively poor preservation, sampling, and dating of earliest Cretaceous dinosaur‐bearing terrestrial exposures, particularly in Gondwana, North America, and Asia (Upchurch *et al.*, 2011; Upchurch & Mannion, 2012; Upchurch, Mannion & Taylor, 2015b; Fig. 5A). The diversity dynamics of the three major dinosaur clades (Ornithischia, Sauropodomorpha and Theropoda) over the J/K boundary appear to have been very different to one another (Barrett *et al.*, 2009; Upchurch *et al.*, 2011) (Fig. 6). Apparent large‐scale changes in the composition of dinosaurian faunas across the J/K boundary led to the original proposal that dinosaurs co‐evolved with the origin and early evolution of flowering plants (Bakker, 1978). This was based on an apparent ecological shift from higher browsing sauropod‐dominated faunas to those composed of more diverse ornithischians. However, numerous recent discoveries in the Late Jurassic to Early Cretaceous interval indicate that such an ecological turnover is not as clearly defined as originally proposed, and the spatiotemporal structure of any such turnover does not support co‐evolutionary relationships between herbivorous dinosaurs and either the origin of angiosperms (Barrett & Willis, 2001; Butler *et al.*, 2009a), or diversificiation of gymnosperms (Butler *et al.*, 2009b).

**Figure 7:**
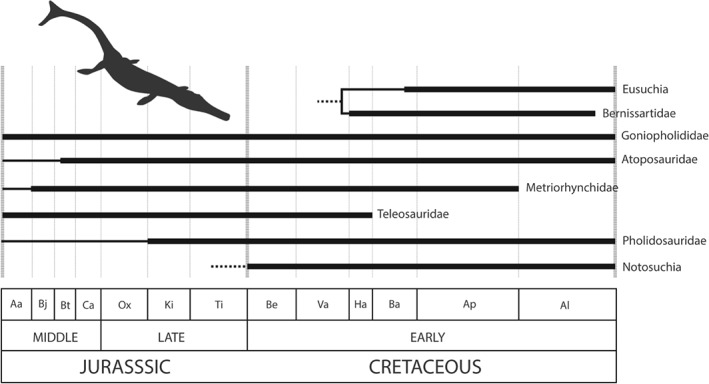
Stratigraphic ranges of major Jurassic–Cretaceous crocodyliform clades. Adapted from Bronzati, Montefeltro & Langer (2012). Dates obtained from *The Paleobiology Database*. Abbreviations as in Fig. 6. Silhouette of *Metriorhynchus geoffroyi* from PhyloPic, by Gareth Monger (CC BY 3.0).

Theropods gradually reduced in diversity through the Late Jurassic, and appear to have been relatively unaffected across the boundary when using a residual diversity estimate based on collection counts (Upchurch *et al.*, 2011; Upchurch & Mannion, 2012). However Lloyd (2012) found evidence of a small decline in diversity, which was also recovered by Upchurch *et al.* (2011) when using a formations‐based residual diversity estimate. This decline is emphasised when birds are excluded, with non‐avian theropod extinction intensity across the J/K boundary reaching a Mesozoic peak (excluding the K/Pg boundary) (Upchurch *et al.*, 2011). This extinction seems to have primarily affected Laurasian faunas (Upchurch *et al.*, 2011; Novas *et al.*, 2013), and appears to have been largely confined to medium‐ to large‐bodied theropods that were more cosmopolitan in nature through the Late Jurassic (e.g. Ceratosauridae, Megalosauridae, and Piatnitzkysauridae), or confined to Euamerica (Allosauridae; Fig. 7A). Other large‐bodied groups, including Carcharodontosauridae and Spinosauridae, have their earliest representatives in the latest Jurassic of Tanzania (Carrano *et al.*, 2012), and preceded the origins and diversification of several major lineages of tetanurans (Carrano *et al.*, 2012; Novas *et al.*, 2013; Zanno & Makovicky, 2013; Tortosa *et al.*, 2014). Smaller‐bodied coelurosaurians (e.g. Troodontidae, Dromaeosauridae) have their origins in the late Middle Jurassic (e.g. Hu *et al.*, 2009; Rauhut, Milner & Moore‐Fay, 2010), but remain largely absent from Early to ‘middle’ Cretaceous Gondwanan theropod faunas, which instead comprise a diverse array of small‐ and large‐bodied taxa (e.g. carcharodontosaurids and spinosaurids) (Sereno *et al.*, 1996; Novas *et al.*, 2005; Brusatte & Sereno, 2007; Evers *et al.*, 2015). Eumaniraptora, or the more inclusive Paraves, underwent a significant acceleration in diversification rates across the J/K boundary (Lloyd *et al.*, 2008). While the first definitive birds first appear in the Late Jurassic of Europe with *Archaeopteryx*, putative members of Aves have also been reported from the Late Jurassic (Oxfordian) of China (Xu *et al.*, 2011; Godefroit *et al.*, 2013; Brusatte *et al.*, 2014). The first major radiation of Aves appears to have occurred in the Early Cretaceous of China (Jehol Biota, Barremian–Aptian), indicated by the diversification of all major pygostylian lineages (O'Connor, Chiappe & Bell, 2011; X. Wang *et al.*, 2014b), although this apparent radiation is likely influenced by the Lagerstätten effect. Furthermore, the earliest Cretaceous fossil record of birds is highly incomplete (Brocklehurst *et al.*, 2012), comprising only fragmentary material (Dyke *et al.*, 2011). This pygostylian radiation was coeval with a rapid diversification of avian bäuplane during the Early Cretaceous (Benson *et al.*, 2014a; Brusatte *et al.*, 2014; M. Wang, O'Connor & Zhou, 2014a), although this was accompanied by an overall constrained ecological disparity (Mitchell & Makovicky, 2014).

Sauropod diversity was high in the Late Jurassic (Kimmeridgian–Tithonian), followed by an apparent dramatic decline over the J/K boundary, based on both raw (Barrett & Upchurch, 2005; Upchurch & Barrett, 2005) and corrected (Mannion *et al.*, 2011; Upchurch *et al.*, 2011; Benson & Mannion, 2012; Lloyd, 2012; Upchurch & Mannion, 2012) estimates of diversity. A similar signal was found by Barrett *et al.* (2009), although they recovered a moderate diversity decline leading up to the J/K boundary, followed by a diversity crash at the boundary. With the possible exception of Spanish taxa whose stratigraphic age cannot be constrained more precisely than late Tithonian–middle Berriasian (Royo‐Torres *et al.*, 2014), non‐neosauropod eusauropods seem to have disappeared at the J/K boundary (Upchurch & Barrett, 2005; Mannion *et al.*, 2011, 2013; Fig. 6B). Late Jurassic representatives of Neosauropoda, comprising Macronaria and Diplodocoidea, were present on all sampled continents except Asia, and this diverse clade crossed the J/K boundary (Upchurch & Barrett, 2005; Mannion *et al.*, 2011). Basal macronarians are only known to have survived into the earliest Cretaceous at least in Europe (Royo‐Torres *et al.*, 2014; Upchurch *et al.*, 2015b) and North America (D'Emic & Foster, in press), although overall titanosauriform diversity was seemingly unaffected across the J/K boundary (Upchurch & Mannion, 2012; Mannion *et al.*, 2013). Cretaceous brachiosaurid diversity appears to have plummeted (or even reduced to zero) outside of North America (D'Emic, 2012; Mannion *et al.*, 2013) and Africa (McPhee *et al.*, 2016), although the clade possibly expanded into the northern tip of South America (Carballido *et al.*, 2015), whilst Somphospondyli experienced a global radiation (D'Emic, 2012; Mannion *et al.*, 2013). Within the narrow‐toothed Diplodocoidea, diplodocids were thought to have gone extinct at the J/K boundary (Upchurch & Barrett, 2005), although recent discoveries in the earliest Cretaceous of Africa (McPhee *et al.*, 2016) and South America (Gallina *et al.*, 2014) indicate that at least one diplodocid lineage survived. Cretaceous dicraeosaurid diplodocoids are also known only from South America (Salgado & Bonaparte, 1991) and Africa (McPhee *et al.*, 2016), whereas rebbachisaurids diversified in northern Africa, Europe and South America in the Early Cretaceous (Carballido *et al.*, 2012).

Ornithischians seem to have been relatively unaffected compared to the other dinosaur groups, with only a moderate decline in diversity at the J/K boundary (Barrett *et al.*, 2009; Upchurch *et al.*, 2011; Fig. 6C). However, the magnitude of any extinction documented by Upchurch *et al.* (2011) is highly dependent on the mode of sampling correction used (i.e. through a collections‐ or formations‐based residual diversity estimate). Diversification rates in Ankylosauria increased rapidly at the J/K boundary (Lloyd *et al.*, 2008) with the North American origin of Ankylosauridae (Arbour & Currie, in press), possibly as they ecologically replaced Stegosauria (the other major group of thyreophoran ornithischians), which were in decline after the J/K boundary, becoming extinct by the end of the Early Cretaceous (Barrett & Willis, 2001; Maidment, 2008). Basal ceratopsians originated in the Late Jurassic (Oxfordian), and were probably unaffected by the J/K boundary (Xu *et al.*, 2006; Benson *et al.*, 2013), and Neoceratopsia may have its origins in the earliest Cretaceous (Valanginian) of Asia (Farke *et al.*, 2014). Ornithopods seem to have been unaffected, with small basal forms proliferating around the J/K boundary (e.g. Han *et al.*, 2012; Escaso *et al.*, 2014; Xing *et al.*, 2014), and iguanodontians becoming increasingly abundant through the Early Cretaceous (Barrett & Willis, 2001).

#### (b) *Pterosaurs*

Most Laurasian pterosaur taxa are known from Konservat‐Lagerstätten, including the Late Jurassic Solnhofen Limestones of southeastern Germany (Wellnhofer, 1970), and the Late Jurassic Daohugou Biota (Sullivan *et al.*, 2014) and late Early Cretaceous Jehol Biota (Wang & Zhou, 2006) of northeastern China. In Gondwana, pterosaur specimens are scarce prior to the late Early Cretaceous (Codorniu & Gasparini, 2013; Fig. 5A), when Lagerstätten such as the Brazilian Crato Formation were deposited (Unwin & Martill, 2007). Initial research into pterosaur diversity patterns hypothesised a peak in diversity at the J/K boundary (Slack *et al.*, 2006). However, more recent analyses have largely overturned this pattern, interpreting diversity peaks to be primarily a product of episodes of enhanced preservation (i.e. the Lagerstätten effect; Butler *et al.*, 2009c, 2013), despite claims that Lagerstätten have little impact on the shape of pterosaur evolution (Dyke *et al.*, 2009).

Pterosaurs underwent a taxonomically selective and staggered extinction phase up to the J/K boundary, with the majority of non‐pterodactyloid pterosaurs (e.g. long‐tailed rhamphorhynchoids) becoming extinct (Unwin, 2003), a pattern that is resilient to the effect of sampling biases (Barrett *et al.*, 2008; Butler *et al.*, 2013; Andres, Clark & Xu, 2014; Upchurch *et al.*, 2015a). Pterodactyloids, particularly ornithocheiroideans, flourished after the J/K boundary, diversifying into a range of species‐rich subclades (Ji, Ji & Padian, 1999; Butler *et al.*, 2013; Andres *et al.*, 2014). Many of these groups originated in the Late Jurassic along with a range of ‘transitional’ species (Liu *et al.*, 2012), but apparently did not radiate until the Cretaceous. Consequently, Late Jurassic and Early Cretaceous pterosaur faunas are quite distinct from one another, although some basal taxa, including the anurognathids (Wang *et al.*, 2009), passed through the J/K boundary.

#### (c) *Crocodylomorphs*

Thalattosuchian crocodylomorphs, comprising two major pelagic groups, Teleosauridae and Metriorhynchoidea, achieved the height of their diversity during the Late Jurassic (Kimmeridgian and early Tithonian, respectively). During this period, thalattosuchians achieved a broad ecological range, with a variety of feeding modes, craniofacial forms, dental morphologies, functional biomechanical behaviours, and a wide spectrum of body sizes (Pierce, Angielczyk & Rayfield, 2009; Andrade *et al.*, 2010; Young *et al.*, 2010, 2011). Geosaurines, a subgroup of metriorhynchoids, possessed a suite of dental characteristics indicating a macrophagous feeding strategy, and it is likely that they were the apex or second‐tier predators of Late Jurassic seas (Andrade *et al.*, 2010; Young *et al.*, 2010, 2011). The other subgroup of metriorhynchoids, metriorhynchines, were smaller and progressively adapted towards an increasingly piscivorous and teuthophagous (squid‐consumption) feeding style towards the latest Jurassic (Young *et al.*, 2011). This ecological dichotomy in metriorhynchids is reflected in their high morphological disparity, although how this changed over the J/K boundary is difficult to discern (Young *et al.*, 2010). Thalattosuchian diversity declined through the J/K boundary based on both raw and subsampled estimates (Mannion *et al.*, 2015), with teleosauroids becoming extinct at the end of the Hauterivian (Fanti *et al.*, in press), and the group had disappeared completely by the end of the Aptian (Young *et al.*, 2014a, 2014b; Martin *et al.*, 2014; Chiarenza *et al.*, 2015; Fig. 7).

Most basal mesoeucrocodylians, including the majority of ‘shartegosuchids’, are known only from the Jurassic, but at least some forms survived into the Cretaceous of Eurasia (Clark, 2011). Metasuchia, the dominant clade within Mesoeucrocodylia, comprises two major clades of crocodylomorphs: the extinct clade Notosuchia, and Neosuchia, which includes Eusuchia and extant Crocodylia. Basal neosuchians, including the exclusively Laurasian semi‐aquatic goniopholids, appear to have passed comparatively unscathed through the J/K boundary (Martin, Rabi & Csiki, 2010; Andrade *et al.*, 2011), although terrestrial atoposaurids seem to have been affected, with Cretaceous occurrences dominated by the shallow marine *Theriosuchus* lineage (Tennant & Mannion, 2014; Young *et al.*, 2016). This pattern of decline is reflected in subsampled diversity estimates of non‐marine crocodyliforms, which decreased through the J/K boundary (Mannion *et al.*, 2015). In the Early Cretaceous, the terrestrial notosuchians diversified, adopting a novel suite of ecophenotypes (Carvalho *et al.*, 2010; O'Connor *et al.*, 2010; Bronzati, Montefeltro & Langer, 2015). Notosuchia may have its origin in the Early Jurassic based on its sister‐taxon relationship with Neosuchia, but the first known occurrence is from the earliest Cretaceous (Berriasian) of Brazil (Carvalho *et al.*, 2010). Notosuchians reached a diversity peak in the Aptian–Albian (Carvalho *et al.*, 2010), but whether this represents the true timing of their early radiation is masked by a poor earliest Cretaceous fossil record (Benson *et al.*, 2013; Fig. 5A). Notosuchians and eusuchians both underwent rapid diversifications in the earliest Cretaceous (Bronzati *et al.*, 2015).

#### (d) *Ichthyopterygians*

Recent analyses have demonstrated that summed marine reptile diversity declined dramatically through the J/K boundary (Benson & Butler, 2011; Kelley & Pyenson, 2015), with evidence that ichthyosaurs were severely affected (Bakker, 1993; Bardet, 1994; Sander, 2000; Benson *et al.*, 2010). However, a range of recent discoveries and taxonomic revisions have challenged this picture, and instead it seems that ichthyosaurs passed through the J/K boundary relatively unscathed (Fischer *et al.*, 2012, 2013a). Nearly all ichthyosaur lineages from the Late Jurassic onwards belong to Ophthalmosauridae. Whereas the ophthalmosaurid subclade Platypterygiinae diversified during the Late Jurassic (Kimmeridgian), only three ophthalmosaurine taxa survived into the Cretaceous with the majority going extinct in the latest Jurassic, and the last known occurrences from the Aptian–Albian of Europe (Zammit, 2012; Arkhangelsky & Zverkov, 2014; Roberts *et al.*, 2014). However, this pattern is obscured by the paucity of earliest Cretaceous ichthyosaur specimens (Fischer *et al.*, 2012; Green & Lomax, 2014; Fig. 5B). *Malawania,* from the Early Cretaceous of Iraq, demonstrates that at least one basal non‐ophthalmosaurid lineage passed through the J/K boundary (Fischer *et al.*, 2013a). Ophthalmosaurines may have been ecologically conservative throughout their evolutionary history, whereas their sister group, Platypterygiinae, exhibited a much broader range of ecological diversity (Fischer *et al.*, 2013b), a factor that might have played a role in their relative macroevolutionary histories. Recent discoveries from the Late Jurassic of Russia (Zverkov *et al.*, 2015) indicate that ophthalmosaurid diversity was high at high latitudes right up until the end of the Jurassic, and that dispersal pathways between Northern and Southern Hemispheres might have been significant in controlling their relatively high survival rates into the Early Cretaceous (Stinnesbeck *et al.*, 2014).

#### (e) *Sauropterygians*

Taxonomic diversity of Plesiosauria declined greatly across the J/K boundary, with the extinction of microcleidid and rhomaleosaurid taxa, known almost exclusively from Euamerica (Bakker, 1993; Benson & Druckenmiller, 2014). Recovery from this extinction did not begin until the Hauterivian–Barremian (Benson & Butler, 2011). The only plesiosaurian taxa that declined immediately prior to the J/K boundary were members of Cryptoclididae, which were restricted to the northern hemisphere [apart from a tentative occurrence from the Kimmeridgian of India (Bardet *et al.*, 1991)], and Pliosauridae, with the exception of the pliosaurid subgroup Brachaucheninae (Ketchum & Benson, 2010; Benson & Druckenmiller, 2014). Four plesiosaurian lineages are known to have crossed the J/K boundary, with the major clades Elasmosauridae and Leptocleididae both diversifying in the earliest Cretaceous (Benson & Druckenmiller, 2014), following a prolonged period of sustained extinction and replacement throughout the Late Jurassic (Benson & Bowdler, 2014). Benson & Druckenmiller (2014) additionally found a substantial decline in plesiosaurian morphological disparity during the Late Jurassic and Early Cretaceous.

#### (f) *Testudinatans*

**Figure 8:**
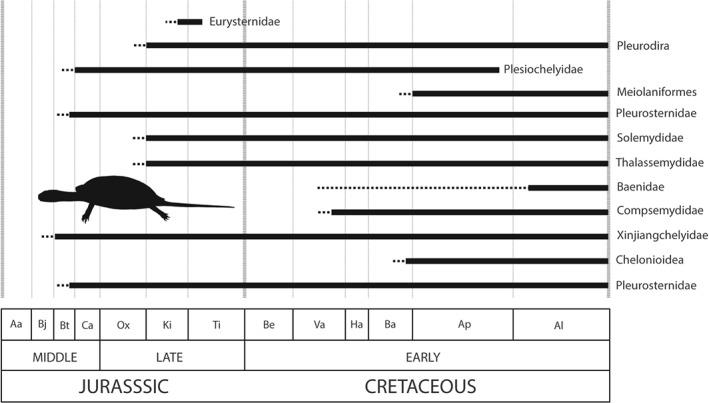
Stratigraphic ranges of major Jurassic–Cretaceous turtle clades. Phylogenetic relationships are not illustrated. Dates obtained from *The Paleobiology Database*. Abbreviations as in Fig. 6. Silhouette of a baenid turtle from PhyloPic, by Scott Hartman (CC BY‐SA 3.0).

The timing of the origin of Testudines, and diversification of its major extant clade Eucryptodira, remains controversial, but both events had occurred by at least the Late Jurassic (Danilov & Parham, 2006; Joyce, 2007; Sterli, Pol & Laurin, 2013). Shallow marine taxa experienced a substantial decrease in diversity across the J/K boundary based on residual diversity estimates (Benson *et al.*, 2010), although Nicholson *et al.* (2015) found only a moderate decline when applying a subsampling approach. However, fully pelagic turtles might not have been present until the origin of Chelonioidea (sea turtles), with the earliest forms known from the Early Cretaceous of South America (Hirayama, 1998; Cadena & Parham, 2015; Fig. 8). Terrestrial turtles, on the other hand, appear to have been largely unaffected (Hirayama, Brinkman & Danilov, 2000), with Nicholson *et al.* (2015) documenting a steady increase in non‐marine diversity through the J/K boundary, peaking in the Aptian. However, this apparent ‘global’ increase is largely driven by data from Europe, with any other continent‐level signal through the J/K boundary poorly resolved (Nicholson *et al.*, 2015). Many Laurasian taxa were endemic in the Late Jurassic, with three major biozones forming in North America, Asia, and Europe (Hirayama *et al.*, 2000). In the Late Jurassic of Europe, an array of eucryptodiran groups, including basal forms, plesiochelyids, thalassemydids, and eurysternids, were abundant and occupied a range of coastal‐marine and freshwater settings (Pérez‐García *et al.*, 2008; Slater *et al.*, 2011; Pérez‐García, de la Fuente & Ortega, 2012; Anquetin & Joyce, 2014; Jansen & Klein, 2014; Pérez‐García, 2014a, 2014b, 2015). Eucryptodires also dominated Asian turtle faunas, whereas those in North America were composed primarily of paracryptodires, including the clades Solemydidae and Pleurosternidae (Hirayama *et al.*, 2000; Lipka *et al.*, 2006; Joyce *et al.*, 2011; Pérez‐García & Ortega, 2014).

Some basal eucryptodirans persisted into the Early Cretaceous of Europe (Pérez‐García *et al.*, 2012), whereas others, such as eurysternids and thalassemydids, became locally extinct in the latest Jurassic (lower Tithonian). Coastal‐dwelling European plesiochelyids might have crossed the J/K boundary based on tentative reports from the Valanginian of Switzerland (Lapparent de Broin, 2001). Only three species of the diverse lineage Pleurosternidae survived into the Cretaceous, which lived alongside paracryptodirans, including the North American Cretaceous groups Baenidae and Compsemydidae (Pérez‐García, Gasulla & Ortega, 2014; Pérez‐García *et al.*, 2015). Groups dominant in Asia, such as the freshwater Xinjiangchelyidae (probable basal eucryptodires), appear to have been unaffected by the J/K boundary (Danilov & Sukhanov, 2006), although there is some evidence suggesting that this group might be paraphyletic, in which case this taxonomic artefact likely masks a notable decline (Rabi, Joyce & Wings, 2010).

In the Early Cretaceous, derived eucryptodiran and paracryptodiran turtles became increasingly diverse in Europe (Pérez‐García, 2012; Pérez‐García *et al.*, 2014; Püntener *et al.*, 2014), following the latest Jurassic extinction of many basal members of these groups. Basal panpleurodirans might have achieved a broad palaeobiogeographic distribution in shallow marine systems during the Late Jurassic (Bardet *et al.*, 2014), followed by the Early Cretaceous diversification of the second major clade of crown group turtles, Pleurodira (Joyce, Parham & Gauthier, 2004; Danilov & Parham, 2008; Cadena, Jaramillo & Bloch, 2013). Platychelyidae, a basal group of freshwater and coastal panpleurodirans from North America, South America, and Europe, went extinct in the Valanginian (Cadena & Joyce, 2015). In Gondwana, Early Cretaceous turtle faunas were dominated by two clades, Pleurodira and Meiolaniformes (Sterli, de la Fuente & Umazano, 2015), the latter first appearing in the Barremian of South America (Sterli, 2015). Of note is the absence of pleurodirans and meiolaniform turtles from Laurasian faunas during the Late Jurassic and Early Cretaceous (Perea *et al.*, 2014).

#### (g) *Choristoderes*

Choristoderes were small‐ to medium‐sized semi‐aquatic middle‐tier predators of Laurasian ecosystems, whose placement within Diapsida remains enigmatic (Ksepka, Gao & Norell, 2005; Matsumoto *et al.*, 2009; Zhou & Wang, 2010). They are relatively rare components of the fossil record, known from only a dozen or so genera, but range through the Middle Jurassic to the Miocene (Evans & Klembara, 2005). The timing of the radiation of the non‐neochoristodere group, Monjurosuchidae, is a point of ongoing study, although their origin might be in the Early Cretaceous of Asia (Gao & Fox, 2005; Averianov *et al.*, 2006; Richter *et al.*, 2010; Gao *et al.*, 2013). The earliest records of the major lineage Neochoristodera occur in Barremian (Early Cretaceous) deposits of Asia (Matsumoto & Evans, 2010) and North America (Britt *et al.*, 2006), and this lineage persisted well past the K/Pg boundary (Evans & Klembara, 2005). There appears to have been an ecological transition around the J/K boundary, from smaller basal forms (Late Jurassic), to larger taxa, primarily representing neochoristoderes (Early Cretaceous onwards), with non‐neochoristoderes seemingly becoming extinct in Euamerica (Matsumoto & Evans, 2010).

#### (h) *Lepidosaurians*

Lepidosauria comprises the diverse and extant groups Rhynchocephalia and Squamata (Evans & Jones, 2010). Numerous diverse lepidosaurian clades originated in the Middle Jurassic and passed through the J/K boundary (Conrad, 2008; Jones *et al.*, 2013), although their diversity dynamics have not been investigated through this period. These ‘Jurassic‐type’ faunas persisted until the Aptian–Albian in North America and Europe, and became increasingly rare as they were replaced by more ‘advanced’ lepidosaur faunas (Evans & Chure, 1999; Nydam & Cifelli, 2002; Nydam, 2013). A pan‐Laurasian fauna was present in the Late Jurassic (e.g. scincoids and anguimorphans), with several of these Laurasian taxa also known from the earliest Cretaceous of North Africa (Richter, 1994; Evans, 2003; Nydam, 2013; Rage, 2013), although Berriasian occurrences are restricted to western Europe, Japan, and North Africa. Late Jurassic Gondwanan occurrences of Lepidosauria are restricted to a single occurrence from Tanzania, identified as a scincomorph squamate (Broschinski, 1999), although Early Jurassic Gondwanan occurrences are also known.

The origins of major extant squamate clades such as Lacertoidea (true lizards), Scincoidea (skinks), and the clade comprising Acrodonta and Pleurodonta (iguanians), are either close to the J/K boundary (Pyron & Burbrink, 2012), or in the Early Cretaceous (Jones *et al.*, 2013; Rage, 2013), based on a combination of molecular and fossil data. The origination time for Serpentes (snakes) is contentious, with some fossil evidence suggesting either the Middle–Late Jurassic (Caldwell *et al.*, 2015) or Early Cretaceous (Martill, Tischlinger & Longrich, 2015), but molecular evidence indicates a younger, early Late Cretaceous age (Head, 2015). The first occurrences of Lacertoidea are in the Berriasian of Western Europe (Evans, Jones & Matsumoto, 2012), before radiating into North America (Nydam & Cifelli, 2002) and Asia (Gao & Cheng, 1999) in the Barremian–Albian. Although lacking a pre‐Cenozoic fossil record, amphisbaenian lacertoids (lizard worms) are thought to have originated around the J/K boundary (Longrich *et al.*, 2015). The earliest known scincoid is from the Albian–Cenomanian of North America (Nydam, 2002). Whereas the earliest fossil acrodont is known from the late Early Jurassic of Asia (Evans, Prasad & Manhas, 2002), the next oldest occurrence is from the Barremian of China (Li *et al.*, 2007). However, pleurodonts, the sister group to acrodonts, are not known until the Late Cretaceous (Norell & Gao, 1997).

Rhynchocephalians are only known from Euamerica in the Late Jurassic, and might have exhibited high ecological diversity, especially with respect to feeding strategy (Rauhut *et al.*, 2012). Their Cretaceous record extends to North Africa (Broschinski, 1999) and South America (Apesteguía & Carballido, 2014). Pleurosauridae represents a small and poorly known basal clade of European marine rhynchocephalians, with a short duration from the Early Jurassic to the early Tithonian (Dupret, 2004; Bardet *et al.*, 2014). Sphenodontia, a group of basal rhynchocephalians, appears to have beeen confined to Euamerica in the Late Jurassic, but radiate into Africa in the Berriasian (Evans & Sigogneau‐Russell, 1997) and South America in the Albian (Reynoso, 2000; Apesteguía & Carballido, 2014).

#### (i) *Lissamphibians*

Lissamphibia comprises anurans (frogs), caudatans (salamanders), albanerpetontids (salamander‐like animals) and gymnophionans (caecilians). Within Lissamphibia, there was a small increase in total diversity over the J/K boundary (Fara, 2004). Molecular dates of lissamphibian radiations have wide uncertainty ranges, but it appears that several species‐rich lineages, particularly within Anura, might have diversified around the J/K boundary (Marjanović & Laurin, 2013). Anurans were largely unaffected across the J/K boundary at higher taxonomic levels, but more work on the systematics of the group is required to clarify its macroevolutionary history (Marjanović & Laurin, 2007). Anurans were diverse in the Late Jurassic of Euamerica, and absent in Gondwana (Evans & Milner, 1993), but in the Early Cretaceous we find their first fragmentary Gondwanan occurrences in South America (Chiappe *et al.*, 1998), Africa (Jacobs *et al.*, 1990), as well as in Asia (Evans *et al.*, 1998). Gymnophionan diversity is unknown across the J/K boundary, with the oldest occurrence in the Sinemurian of North America (Jenkins & Walsh, 1993), and the next and only Early Cretaceous occurrence from the Berriasian of Morocco (Evans & Sigogneau‐Russell, 2001).

In the Late Jurassic of North America, the lissamphibian fossil record documents a mixture of stem caudates and anurans, as well as the first North American crown caudate (Henrici, 1998; Evans *et al.*, 2005; Gardner & DeMar, 2013). There might be a ‘hidden’ Late Jurassic diversity in North America, as Gardner & DeMar (2013) indicated that there could be as many as five unnamed anuran species, and an additional unnamed caudate, in the Quarry 9 and Rainbow Park localities alone. The Late Jurassic to Early Cretaceous record of central and western Asian salamanders and albanerpetontids is poor, with only a single stem salamander species known (Ivakhnenko, 1978; Skutschas, 2013). Salamanders from China are currently reforming our understanding of their Late Jurassic to Early Cretaceous evolution, but different views on the dating of associated beds make constraining their ages, and the timings of important radiations, problematic (Wang & Evans, 2006). Several of these taxa occupy basal positions within Caudata, suggesting that events around the J/K boundary in eastern Asia might have been important in their early evolution (Wang, 2000; Zhang *et al.*, 2009). The dynamics of salamander diversification in Laurasia remain obscured by fragmentary specimens (Evans *et al.*, 2005), but the two major extant clades, Salamandroidea and Cryptobranchoidea, both appear to have passed through the J/K interval unperturbed. It is unknown when or where the split of Cryptobranchoidea into two large subclades, Hynobiidae and Cryptobranchidae, took place (Gao & Shubin, 2003), although major extant sublineages have been radiating since at least the Early Cretaceous (Gao & Shubin, 2001). Until recently, no stem salamanders were known from post‐J/K boundary deposits, suggesting that they went extinct between the Kimmeridgian and end‐Jurassic (Skutschas, 2013), but a new relict taxon from the Aptian–Albian of Siberia suggests that they survived in an isolated refugium (Skutschas, in press). Albanerpetontids appear to have been confined to western Europe during the Late Jurassic, prior to radiating into Africa during the Berriasian (Gardner, Evans & Sigogneau‐Russell, 2003), and into North America during the Aptian–Albian (Cifelli *et al.*, 1997; Gardner, 1999).

#### (j) *Mammaliaforms*

The Late Jurassic was an important time in the rise of modern‐day mammal clades, with the diversification of Theria (comprising Eutheria and Metatheria) occurring around 160 Ma, during the Oxfordian (Luo *et al.*, 2011; Williamson, Brusatte & Wilson, 2014). Members of Theria remained relatively rare (although morphologically derived; see, for example, Sigogneau‐Russell, 1998) through the J/K interval and into the Early Cretaceous. A recent analysis, however, placed these Jurassic occurrences outside of Theria (Krause *et al.*, 2014), implying that the earliest known occurrences of this group were in the Barremian (Ji *et al.*, 2002; Luo *et al.*, 2003). Only a single Gondwanan mammaliaform occurrence is known from the Late Jurassic of Africa (Tanzania; Dietrich, 1927), which is quite different to the more diverse earliest Cretaceous (Berriasian) African fauna (e.g. from Morocco; Sigogneau‐Russell, 1995, 1999).

**Figure 9:**
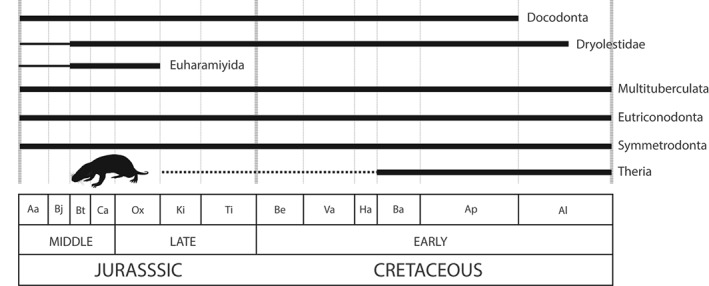
Stratigraphic ranges of major Jurassic–Cretaceous mammaliaform lineages. Dates obtained from *The Paleobiology Database*. Abbreviations as in Fig. 6. Silhouette of *Moragnucodon watsoni* from PhyloPic, by FunkMonk (CC BY‐SA 3.0).

All major Late Jurassic mammalian clades persisted into the Early Cretaceous (Fig. 9), including an array of forms such as basal cladotherians, multituberculates, triconodonts, and symmetrodontans, as well as rarer non‐mammalian synapsids in Russia and Japan (Kielan‐Jaworowska, Cifelli & Luo, 2004; Zheng, Wang & Meng, 2013). Although no significant clades went extinct at the J/K boundary, more advanced mammalian groups (including multituberculates and eutriconodonts) displaced more primitive and contiguous mammaliaform lineages (i.e. dryolestids and docodonts) during the Late Jurassic, with docodonts surviving until at least the Berriasian of the UK (Cifelli, Davis & Sames, 2014), and perhaps even later in Russia (Averianov & Lopatin, 2015). Cifelli *et al.* (2014) suggested that this pattern represents a geographically constrained and gradual taxonomic turnover at the onset of the Cretaceous, as evidenced by occurrences of tribosphenidans in North America and Europe (Cifelli & Davis, 2015), but with their absence in Russia along with multituberculates being explained by an abundance of tritylodontids (Averianov *et al.*, 2015). This conclusion was reinforced by Newham *et al.* (2014), who found that global mammaliaform diversity either dropped through the J/K boundary (using a residuals method), or increased slightly (using SQS), with little change from ‘Jurassic‐type’ faunas over the boundary, whereas North American diversity shows a decline.

Within therians, or closely related forms, a broad array of ecophenotypes diversified in the Late Jurassic, including scansorial, fossorial, insectivorous, carnivorous, gliding, and swimming forms, all with small body masses (<12 kg; Luo & Wible, 2005; Luo, 2007; Zheng *et al.*, 2013). In multituberculates, disparity declined through the J/K boundary, and mean body size remained constant (Wilson *et al.*, 2012), coincident with generally low rates of phenotypic evolution and low disparity of mammals in the Late Jurassic to Early Cretaceous transition (Close *et al.*, 2015). This was paralleled by a burst in taxonomic richness immediately preceding the boundary, followed by a minor fall in the Early Cretaceous (Wilson *et al.*, 2012), and the acquisition of key multituberculate characteristics (Yuan *et al.*, 2013).

#### (k) *Fish groups*

Early studies found either a small increase (Benton, 1993) or small decrease (Carroll, 1988) in fish diversity over the J/K boundary. Friedman & Sallan (2012) used Sepkoski's (2002) compendium to demonstrate that marine fishes experienced extinction rates across the J/K boundary of the same magnitude as those of the K/Pg boundary, coupled with reduced origination rates. This magnitude is relatively depressed when looking exclusively at marine chondrichthyans, but in marine osteichthyans the J/K extinction rate is higher than that for the K/Pg (Friedman & Sallan, 2012). This increased extinction rate at the J/K boundary coincides with one of two peaks in taxic diversity (the Late Jurassic and early Late Cretaceous), which has been attributed to increased preservation episodes (Friedman & Sallan, 2012). However, Lloyd & Friedman (2013) found little evidence for a significant change in fish diversity through the J/K boundary, although data are absent for many sampling‐corrected curves in the Late Jurassic in this study. Actinopterygian diversity appears to be stable through the J/K boundary, although there is a diversity shift from marine to freshwater taxa (Cavin, Forey & Lécuyer, 2007). Teleost fish radiated during the Late Jurassic to Early Cretaceous, replacing many of their holostean‐grade predecessors (e.g. Steel, 1973).

Neoselachia, the clade including all modern forms of shark, and Batoidea (skates and rays) both underwent a phase of high diversification rates during the latest Jurassic (Kriwet, 2003; Rees, 2005). This was followed by a species‐level diversity decline at the J/K boundary, resulting from decreased origination rates and heightened extinction rates (Kriwet & Klug, 2008; Kriwet, Kiessling & Klug, 2009a). However, no major neoselachian clades went extinct at the J/K boundary, and their Early Cretaceous standing diversity was substantially higher than Late Jurassic levels (Underwood, 2006; Kriwet *et al.*, 2009a; Kriwet, Nunn & Klug, 2009b; Guinot, Adnet & Cappetta, 2012; Klug & Kriwet, 2013). A recent study showed that nearly all major extant lineages of sharks were already present in the latest Jurassic or earliest Cretaceous, with the origins of Squaliniformes, Squatiniformes, Orectolobidae, Lamniformes, and Carchariniformes occurring immediately prior to the boundary, and the timing of diversification of multiple important sublineages intimately associated with the J/K boundary (Sorenson, Santini & Alfaro, 2014).

### (4) Invertebrates

As with vertebrate groups, early analyses of raw (uncorrected) taxic invertebrate diversity have been superseded by global data sets and advanced analytical subsampling approaches (e.g. Foote, 2000; Alroy *et al.*, 2001, 2008; Bush & Bambach, 2004; Alroy, 2008, 2010a, 2010b, 2014). For all marine palaeofaunas, Alroy *et al.* (2001) recovered a diversity trough at the J/K boundary, but at a range of intensities depending on the method used to correct for sampling biases. This result was confirmed by Alroy *et al.* (2008) and Alroy (2010a) in both ‘modern‐type’ and ‘Palaeozoic‐type’ faunas (*sensu* Sepkoski, 1981), with the former experiencing the greatest diversity drop of the two; however, Alroy (2010b) found no such decline. In his most recent analysis, Alroy (2014) detected a peak in extinction rates for all marine invertebrate taxa at the J/K boundary, similar in magnitude to that of the Silurian–Devonian boundary. A range of additional studies recovered this signal of decreasing diversity through the J/K boundary (e.g. Peters & Foote, 2001; Lu, Yogo & Marshall, 2009; Smith, Lloyd & McGowan, 2012). Many of these analysed the dynamics of marine invertebrate faunas as a whole, rather than for individual geographical regions. As such, it is possible that this global diversity decline is a product of different regional‐level patterns, with declines focused primarily in North America, Chile, and Europe (Smith & McGowan, 2007; McGowan & Smith, 2008; Rogov *et al.*, 2010). Alroy (2010b) also found geographical variation, recovering a more severe diversity drop in the northern hemisphere. In addition, many of these studies have grouped invertebrates together, rather than examine patterns in individual clades (although see Alroy, 2010b), which could potentially mask clade‐specific variation.

#### (a) *Molluscs*

Both raw and subsampled bivalve generic diversity declined at the J/K boundary (Skelton *et al.*, 1990; Jablonski *et al.*, 2003; Alroy, 2010b), coincident with increasing phylogenetic clustering of extinction (Roy, Hunt & Jablnoski, 2009). This drop is most pronounced in heteroconch and lucinoid bivalves, but is not as dramatic in other taxa, such as arcoids and pteriomorphs (Roy *et al.*, 2009). These extinctions might have been greater in taxa that inhabited shallow, rather than deeper water environments (Zakharov & Yanine, 1975), although more recent studies have not replicated this result (Skelton *et al.*, 1990). Endemic faunas became depleted in the southern hemisphere over the J/K boundary, except in the southern Andes and East Africa (Damboreana, 2002). A faunal transition zone, created by a strong Tethyan influence, existed between more northern regions (such as India, Arabia and northeast Africa) and southern faunas during the J/K interval (Kauffman, 1973; Damboreana, 2002). Together, these factors suggest that a combination of tectonics, geography, and palaeoceanography exerted a strong control on bivalve diversity and distribution over the J/K boundary.

Early studies of ammonite diversity found evidence of a minor faunal turnover at the J/K boundary, with diverse Tethyan groups such as Perisphinctidae being replaced by Berriasellidae and Spiticeratinae (Sandoval, O'Dogherty & Guex, 2001). Ammonite standing diversity varied greatly around the J/K boundary, with a substantial trough in diversity continuing into the Early Cretaceous (Vinarski, Bondarev & Markov, 2011). Ammonite faunas are thought to have undergone a period of fluctuating provinciality over the J/K boundary, in concert with elevated speciation rates (Raup & Boyajian, 1988; Riccardi, 1991; Cecca *et al.*, 2005; Rogov *et al.*, 2010), leading to the diversification of several new ammonite lineages (Cecca, 1997, 1998, 1999). More recent explorations of diversity patterns, for all cephalopods, show a constant and severe decline in generic richness throughout the Late Jurassic, with diversity reaching a minimum in the Early Cretaceous before beginning to recover (Alroy, 2010b). Regionally, declines in ammonite diversity at the J/K boundary have been reported from South America and Madagascar (although this might instead relate to the opening of the South African seaway between the Tethys and South Pacific oceans; Riccardi, 1991), and from India (Bardhan *et al.*, 1989; Bardhan, Shome & Roy, 2007; Shome & Bardhan, 2009). However, the record from southeast Africa and Australasia over this period is too poor (e.g. Crame, 2002) for a pan‐Gondwanan extinction event to be inferred.

At a raw taxonomic level, gastropod diversity appears to have been unaffected at the J/K boundary (Vinarski *et al.*, 2011). However, sampling‐corrected generic diversity shows a decline at the J/K boundary to a level almost as low as at the Triassic/Jurassic (Tr/J) boundary (Alroy, 2010b).

#### (b) *Brachiopods*

Early studies found little evidence for a significant drop in brachiopod diversity at the J/K boundary (Ager, 1975; Hallam, 1986; Prosorovskaya, 1993). However, Alroy (2010b) recovered a moderate diversity drop at the J/K boundary using subsampling methods, of equal magnitude to the Tr/J boundary extinction, although whether this truly represents a global pattern is yet to be fully explored (e.g. Ruban, 2006; Curry & Brunton, 2007). Some evidence suggests that species‐level diversity declined locally by up to 75% (e.g. in the Northern Caucasus), reflected in the loss of supraspecific taxa, a decrease in the rate of originations, and an increase in extinction rates (Ruban, 2006, 2011). This might be a reflection of latitudinal constraints on brachiopod distribution, with taxa largely restricted to low northern latitudes over the J/K boundary (Powell, 2009; Naimark & Markov, 2011). Terebratulids appear to have diversified as rhynchonellids declined at the J/K boundary, whereas there is a much more marked decline in observed terebratulid diversity than that of rhynchonellids during the Early Cretaceous, on both a regional and global scale (Vörös, 2010; Ruban, 2011).

#### (c) *Reefs*

Coral diversity appears to have increased linearly through the J/K boundary based on subsampled estimates (Alroy, 2010b); however, Kiessling (2008) found a substantial reef expansion in the early Late Jurassic, followed by a comparable decline in the latest Jurassic and over the J/K boundary (see also Kiessling, Aberhan & Villier, 2008). In the Late Jurassic, low‐latitude shallow marine regions were dominated by scleractinian coral reefs (Leinfelder, 2001; Martin‐Garin, Lathuliere & Geister, 2012), with sea level exerting a strong control on their regional distribution (Bambach, 2006). In the Early Cretaceous, there was a shift towards rudist‐dominated reef colonies in shallow environments (Scott, 1988, 1995). The precise timing of this scleractinian‐to‐rudist turnover is poorly constrained, but potentially relates to environmental changes during the Barremian (Scott, 1995; Hofling & Scott, 2002; Gotz, Loser & Schmid, 2005), with Late Jurassic and earliest Cretaceous (Berriasian–Valanginian) faunas remaining compositionally consistent (Gotz *et al.*, 2005). Extremely high extinction and origination rates in scleractinian corals in the latest Jurassic might play a role in this faunal turnover, but they could also possibly relate to different environmental and/or preservational regimes in the Late Jurassic and Early Cretaceous (Simpson *et al.*, 2011).

#### (d) *Echinoderms*

The impact of sampling on global patterns of pre‐Cretaceous echinoderm diversity has yet to be explored in a manner similar to that of other marine invertebrate groups (Alroy, 2010b), hindering our understanding of their dynamics over the J/K boundary. At both the species and family level, raw global echinoderm standing diversity increased from the Late Jurassic to Early Cretaceous, with diversity at an ‘intermediate’ level with respect to overall Phanerozoic diversity patterns (Raup, 1975; Markov, Bondarev & Vinarsky, 2012). Echinoderms might have experienced ecologically selective perturbations through the Late Jurassic and over the J/K boundary (Aberhan, Nürnberg & Kiessling, 2012), although no major lineages went extinct at or around the boundary (Kroh & Smith, 2010); instead, there is evidence for the origins of major clades comprising multiple extant lineages occurring during the Early Cretaceous (Kroh & Smith, 2010). During the earliest Cretaceous, there is some evidence of low regional diversity (e.g. in European localities), before an increase in the Aptian (Smith & Benson, 2013; Pereira *et al.*, 2015). Disparity and diversity appear to have been decoupled in several geographically widespread boundary‐crossing echinoderm lineages (Atelostomata, Disasteroidea), with a marked decline in disparity in the latter group coincident with a geographic range restriction to Europe (Eble, 2000). Uncorrected (‘raw’) crinoid diversity also declined through the J/K boundary, culminating in an extinction peak in the earliest Cretaceous (Gorzelak *et al.*, 2015).

#### (e) *Arthropods*

All major hexapod groups passed through the J/K boundary (e.g. Grimaldi, 2010; Nicholson, Ross & Mayhew, 2014), although their diversity dynamics are poorly understood at lower taxonomic levels. Total family‐level insect diversity declined in the latest Jurassic, subsequent to a sharp increase in their diversity (Labandeira, 2005). There was a Late Jurassic spike in origination rates that can be partially attributed to enhanced episodes of preservation (e.g. the Karatau deposits, Kazakhstan; Ponomarenko, 1988; Labandeira & Eble, in press), but also reflects a terrestrial revolution in insect faunas, with the diversification of phytophagous and parasitoid taxa (Labandeira & Currano, 2013). This radiation was accompanied by a major extinction peak in insect families throughout the Late Jurassic, which might have resulted from competitive displacement of less‐derived insect faunas (Labandeira, 2005).

Multiple derived insect clades (e.g. Hemiptera, Coleoptera, Diptera, and Hymenoptera) appear to have increased dramatically in diversity in the Late Jurassic (Kimmeridgian), followed by a burst of intra‐family diversification during the Early Cretaceous (Labandeira & Sepkoski, 1993; Sohn, Labandeira & Davis, 2015; Labandeira & Eble, in press). Origination rates at the family level in Apterygota increased after the J/K boundary, whereas the four other major insect groups (Palaeoptera, Polyneoptera, Paraneoptera, and Holometabola) experienced slightly depressed origination rates at the boundary, with no notable overall changes in extinction rates (Nicholson *et al.*, 2014). Major groups of Lepidoptera might have emerged in the Late Jurassic–Early Cretaceous interval, paving the way for them to become one of the most diverse insect groups today (Connor & Taverner, 1997; Kristensen & Skalski, 1998; Sohn *et al.*, 2015). Overall coleopteran diversity appeared to increase through the J/K boundary (Smith & Marcot, 2015), and there is some evidence that the fragmentation of Gondwana led to the diversification of major lineages in the earliest Cretaceous (Kim & Farrell, 2015).

Myriapods are poorly known from the Jurassic and Cretaceous, but at least one group, Geophilomorpha, is known to have originated in the Late Jurassic, and no lineages are known to have gone extinct at the J/K boundary (Shear & Edgecombe, 2010). Whether or not chelicerates were affected at the J/K boundary is currently unknown, but extant families of scorpion (Chactidae, Hemiscorpiidae) have their first occurrences in the Early Cretaceous of South America (Dunlop, 2010), and some diverse lineages of spiders, including Juraraneidae, might have undergone rapid diversification events at the J/K boundary (Penney, 2004).

Decapod diversity suffered a dramatic decline over the J/K boundary in all three main groups (true crabs, hermit crabs, and lobsters and shrimp; Klompmaker *et al.*, 2013). From the Late Jurassic to the Early Cretaceous, there was a replacement of highly diverse basal brachyuran (crab) lineages, such as Homolodromioidea, by other species‐rich lineages, including Raninoidea and Calappoidea (Förster, 1985; Luque, 2015). This was accompanied by an environmental shift from reef‐dwelling taxa to those preferring muddier, deeper, and colder waters (Krobicki & Zatoń, 2008).

#### (f) *Bryozoans*

Late Jurassic and Early Cretaceous bryozoan faunas were almost entirely comprised of cyclostome stenolaemates (Taylor & Ernst, 2008), which reached their lowest post‐Triassic diversity in the Tithonian (Taylor & Waeschenbach, 2015). Cheilostomes represent the largest group of extant bryozoans, and first occur in the fossil record in the Late Jurassic (Taylor, 1994). However, their diversification appears to have been constrained until the late Early Cretaceous (Taylor & Waeschenbach, 2015).

### (5) Microfossils

In the Early Cretaceous, microfossil groups became the most volumetrically significant biogenic constituent of deep‐sea sediments for the first time (Hart, 1999; Tremolada *et al.*, 2006; Lukeneder *et al.*, 2010; Pruner *et al.*, 2010; Olivier *et al.*, 2012).

#### (a) *Foraminifera*

Global studies indicate that foraminiferan standing diversity was not affected at the J/K boundary, but that extinction rates in the Middle–Late Jurassic were of equal magnitude to the ‘Big Five’ mass extinctions, and accompanied by high origination rates (Kaminski, Setoyama & Cetean, 2010). This shifted to a regime of depressed origination and extinction rates after the J/K boundary (Kaminski *et al.*, 2010). Regional‐scale studies indicate that foraminiferan species were in a state of geographical flux through the J/K boundary (Rogov *et al.*, 2010), indicating that diversity declined in a spatially controlled manner (Ruban, 2010, 2011).

#### (b) *Radiolarians*

Radiolarians experienced declining diversification rates throughout most of the Late Jurassic, in concert with a dramatic fall in their diversity (Kiessling, 2002). This shifted to an increase in origination rates and diversity in the late Tithonian and Early Cretaceous (Danelian & Johnson, 2001; Kiessling, 2002; Grabowski *et al.*, 2013), although Kocsis, Kiessling & Pálfy (2014) recovered strongly depressed origination rates at the end of the Jurassic, and no significant changes in diversity through the J/K boundary. In relative terms, the J/K boundary saw three times as many boundary‐crossing radiolarian genera as the Tr/J boundary, with Jurassic and Cretaceous faunas remaining largely unchanged (O'Dogherty *et al.*, 2009).

#### (c) *Plankton*

The J/K boundary saw a global revolution in calcareous phytoplankton, with a distinct impact on marine geochemical cycles and carbonate sedimentation (Bralower *et al.*, 1989; Bornemann *et al.*, 2003; Falkowski *et al.*, 2004; Weissert & Erba, 2004; Tremolada *et al.*, 2006; Wimbledon *et al.*, 2011). As noted above (Section (2), many studies demonstrate that δ^13^C values decreased through the J/K boundary; rather than being indicative of increased oceanic productivity, such isotopic trends are typically associated with decelerating hydrological cycling and increasingly oligotrophic conditions (e.g. Weissert & Channell, 1989), despite global changes in calcareous phytoplankton production. Some estimates place the rate of extinction in calcareous nannoplankton at the J/K boundary at five times higher than that of background rates (Roth, 1989; Bown, Lees & Young, 2004), whereas the middle–late Tithonian saw significant radiations in both coccolithophores and nannoliths (Erba, 2006), with enhanced rates of speciation (Bown *et al.*, 2004; Săsăran *et al.*, 2014) and extinction (Lloyd *et al.*, 2012) occurring in both groups at the J/K boundary. It is possible that these events are related to the Early Cretaceous diversification of diatoms and grasses (Falkowski *et al.*, 2004).

### (6) Plants

Cascales‐Miñana & Cleal (2013) recently demonstrated that the ‘Big Five’ mass extinctions are not reflected in the record of vascular plants, and that there is no evidence for significant change at the J/K boundary (see also Cleal & Cascales‐Miñana, 2014). The J/K boundary was also not identified as a mass‐extinction event in plants by McElwain & Punyasena (2007). In terms of higher‐level diversity, pteridophytes were a relatively minor component of terrestrial ecosystems; instead, environments on land were dominated by gymnosperms, before angiosperms began their ascent in the Early Cretaceous (e.g. Niklas, 1988; Philippe *et al.*, 2008; Coiffard *et al.*, 2012), possibly driven by tectonically influenced changes to atmospheric carbon levels and climate change (Barrett & Willis, 2001; Chaboureau *et al.*, 2014), or increasing environmental disturbance of angiosperm environments by herbivores (Barrett & Willis, 2001). However, in the earliest Cretaceous, floras were still dominated by cycadophytes, ferns, and conifers (Butler *et al.*, 2009a, 2009b), with angiosperms absent from pre‐Hauterivian‐aged rocks, and there were no significant changes in the abundance of major plant groups across the J/K boundary (Barrett & Willis, 2001).

During the Jurassic, floral diversity and productivity were highest at mid‐latitudes, due to the migration of productivity concentrations during greenhouse episodes (Rees, Zeigler & Valdes, 2000). In North America, the Kimmeridgian–Tithonian was a period of humid climates, echoed in the preserved floral diversity (Parrish, Peterson & Turner, 2004). In Eurasia we see a shift from Cheirolepidiaceae‐dominated forests to a high‐diversity palynoflora composed of other conifers, cycads and pteridophytes (Abbink *et al.*, 2001; Zhang *et al.*, 2014), reflecting a change from a drier to a more humid climate over the J/K boundary. Important groups such as the aquatic clavatoracean charophytes were restricted to the Central Tethyan Archipelago throughout the late Tithonian to early Berriasian (Martín‐Closas, Sames & Schudack*,* 2013). The complex palaeogeography of Europe at this time led to enhanced allopatric speciation and isolation of plants from nearby continents, with dispersal to Asia and North America initiated in the late Berriasian to early Valanginian (Martín‐Closas *et al.*, 2013).

The South Pole and high latitudes were dominated by polar forests largely comprising podocarps and araucarians during the Early Cretaceous (Douglas & Williams, 1982; Dettmann, 1989). High‐latitude terrestrial regions of the southern hemisphere had similar forests from the latest Jurassic through to the close of the Cretaceous, with a steep floral zonation gradient (Dettmann, 1989). Floral groups in Australia (and associated landmasses) and Antarctica appear to have been unaffected at the J/K boundary, although, based on currently available data, any species‐level effect is unknown (Dettmann, 1989).

## IV. DISCUSSION

### (1) Evidence for a mass extinction

Whether or not there was a mass extinction at the J/K boundary is a multi‐faceted issue, and occluded by the relatively poor sampling and dating of earliest Cretaceous fossil‐bearing deposits (Fig. 5), as well as the different approaches used in its historical investigation. There has been a great range, both in scope and method, in the way in which analytical techniques have been applied to address the issues of heterogeneous sampling regimes at this time and, as such, providing a single accepted figure or range for extinction intensity at the J/K boundary is problematic. However, what can be identified are a series of group‐specific changes at and around the J/K boundary, including lineage terminations and faunal turnovers, along with potentially associated environmental perturbations. There are documented drops in diversity in both the marine and terrestrial realms, at different scales and in different groups, that together point towards an event that warrants more detailed investigation, particularly in the context of sampling standardised diversity curves and the teasing apart of biotic and abiotic drivers of the resulting patterns (e.g. Feulner, 2011; Wall, Ivany & Wilkinson, 2011; Mayhew *et al.*, 2012; Alroy, 2014).

Vertebrate groups such as theropod and sauropod dinosaurs, rhamphorhynchid pterosaurs, marine crocodylomorphs and testudines, sauropterygians, and groups of fishes, all show evidence of a decline in diversity across the J/K boundary. However, in almost all of these cases, total net diversity of their more inclusive higher clade remained high, with heightened rates of speciation accompanying elevated rates of extinction. Within invertebrates, reef‐dwelling taxa, including corals and some arthropod groups, were the primary victims across the J/K boundary. Furthermore, ammonites, gastropods, brachiopods, foraminiferans, and calcareous phytoplankton all have documented drops in diversity or increased extinction rates at the J/K boundary. Currently, however, the evidence from the fossil record indicates that the J/K boundary cannot be regarded as a mass extinction of the same magnitude as the ‘Big Five’.

### (2) Abiotic factors influencing diversity

#### (a) *Terrestrial patterns*

There is some evidence for geographic selectivity of extinction in theropods through the J/K boundary, with the differentiation between coelurosaurian and non‐coelurosaurian‐dominated theropod faunas within Gondwana and Laurasia, respectively (e.g. Benson *et al.*, 2013). However, although this might reflect a genuine spatial and taxonomic signal, with extinction and diversification events affecting major theropod lineages differently (Upchurch *et al.*, 2011), this could alternatively be a product of different taphonomic conditions (i.e. the lack of Lagerstätten in Gondwana). A similar pattern is evident in sauropods, with Euamerican taxa appearing to suffer a greater magnitude of extinction than their Gondwanan counterparts (Upchurch *et al.*, 2011). This signal is further reflected in smaller‐bodied tetrapod groups, with mammaliaforms showing evidence of a Euamerican diversity decline, in a period of otherwise stable global diversity (Newham *et al.*, 2014). The fact that this extinction varies on a geographic scale, with Asian and African taxa seemingly unaffected, implies two possibilities: firstly, that this signal is a product of heterogeneous spatial sampling regimes; and/or secondly, that this is a result of different geographical controls on extinction across the J/K boundary.

There might also be a degree of facies‐ or environmentally oriented selectivity in turtles during the Late Jurassic and Early Cretaceous. Only a single freshwater taxon is currently known to have survived the J/K boundary, among an assortment of European coastal and semi‐aquatic plesiochelyids, eurysternids, and thalassemydids (Pérez‐García & Ortega, 2014; Cadena & Joyce, 2015), although xinjiangchelyids might have persisted in a European Cretaceous refugium (Pérez‐García *et al.*, 2014). Similarly in Asia, it is likely that palaeoenvironmental preferences and seasonal climatic variations controlled turtle distributions through the J/K boundary and until the late Early Cretaceous (Rabi *et al.*, 2010). Whether or not this pattern is reflected in crocodylomorphs – the only other tetrapod group to have terrestrial and fully marine forms at this time – is currently unknown, although it does appear that semi‐aquatic groups, such as atoposaurids, were particularly affected across the J/K boundary.

Geographic selectivity might have played a role in the diversification of plants, with the development of endemism in some groups, followed by dispersal‐induced cosmopolitanism, a pattern that might be characteristic of both floras and faunas through the progressive break‐up of Pangaea (e.g. Martín‐Closas *et al.*, 2013). It is likely that shifting climates strongly influenced floral diversity on a regional scale in the Late Jurassic (e.g. Dettmann, 1989; Rees *et al.*, 2000; Parrish *et al.*, 2004), but diversity patterns for major groups across the J/K boundary are currently unknown.

#### (b) *Marine patterns*

A faunal turnover is documented in sauropterygian taxa, along with the extinction of shallow marine and semi‐aquatic crocodylomorphs and testudines at the J/K boundary (Benson *et al.*, 2010; Benson & Butler, 2011; Benson & Druckenmiller, 2014; Martin *et al.*, 2014). The staggered Late Jurassic decline in thalattosuchians, along with semi‐aquatic crocodylomorphs, is likely to have been related to the closing off of shallow marine basins during a global sea‐level regression (Hallam, 1988, 1992, 2001; Miller *et al.*, 2005; Pierce *et al.*, 2009). It might be that among marine tetrapods, those fully adapted to a free‐swimming lifestyle (such as ichthyosaurs) were more resistant to regional sea‐level changes occurring over the J/K boundary by exploiting new dispersal pathways (Zammit, 2012; Stinnesbeck *et al.*, 2014; Zverkov *et al.*, 2015), and therefore did not experience elevated extinction rates.

Articulate brachiopods, gastropods, bivalves and ammonites all declined in diversity at the J/K boundary, with the latter two groups showing evidence for regional selectivity (Alroy, 2010a; Rogov *et al.*, 2010), alongside a higher extinction intensity in northern hemisphere taxa (Alroy, 2010b). Additionally, these groups exhibited latitudinal constraints on diversity, possibly driven by large‐scale changes in global climate regimes at the J/K boundary (Anderson *et al.*, 1999; Scotese *et al.*, 1999; Bergman *et al.*, 2004; Meyers, 2014). Such constraints might be responsible for global declines in diversity (Peters & Foote, 2001; Smith & McGowan, 2007; McGowan & Smith, 2008; Lu *et al.*, 2009; Alroy, 2010b; Rogov *et al.*, 2010; Valentine & Jablonski, 2010; Smith *et al.*, 2012), and the reorganisation of marine ecosystems through the J/K boundary. Additional evidence suggests that climate strongly influenced oceanic productivity and nutrient cycles at the J/K boundary (Danelian & Johnson, 2001), which, combined with a eustatic lowstand (Miller *et al.*, 2005), would have strongly impacted upon marine life. This could have provided a mechanism for the different patterns exhibited by shallow‐ and deep‐water invertebrate taxa. There is a growing body of evidence that low‐latitude reef‐dwelling or shallow‐marine and sessile epifauna (e.g. cemented bivalves, corals) were the most severely affected at the J/K boundary (Zakharov & Yanine, 1975; Skelton *et al.*, 1990; Aberhan *et al.*, 2006; Kiessling, 2008, 2009; Alroy, 2010a; Ruban, 2011; Foote, 2014), possibly due to a dramatic shift from calcitic to aragonitic organisms (Kiessling *et al.*, 2008).

The decline of reefs over the J/K boundary was probably also tied to changes in global temperatures (Anderson *et al.*, 1999; Scotese *et al.*, 1999; Bergman *et al.*, 2004; Martin‐Garin *et al.*, 2010). Additionally, it is likely that factors relating to sea‐level changes, including declining salinity and shifts in nutrient flux systems, constrained organisms to increasingly rare shallower shelf systems over the J/K boundary and until the middle Cretaceous (Hay *et al.*, 2006). The core driver for these changes in sea level and marine productivity (Danelian & Johnson, 2001) potentially relates to the connection between the Atlantic and the Pacific (Panthalassa) oceans during the J/K interval, with shorter term variation driven by fluctuations in the extent of polar ice caps (Haq, 2014). Large‐scale tectonic processes at this time, particularly regarding the break‐up of Pangaea, must have been important in controlling the biogeography of marine and terrestrial taxa through the latest Jurassic to earliest Cretaceous (Galton, 1982; Scotese *et al.*, 1988; Scotese, 1991; Pérez‐Moreno *et al.*, 1999; Mateus, 2006; Escaso *et al.*, 2007). However, whether sea level is alone in driving these documented extinctions, or whether dramatic climatic changes played a role (Anderson *et al.*, 1999; Scotese *et al.*, 1999; Bergman *et al.*, 2004; Meyers, 2014) is presently unknown.

#### (c) *Additional environmental changes that require exploration*

There is substantial evidence for a major sea‐level regression at the J/K boundary (Haq *et al.*, 1987; Miller *et al.*, 2005; Fig. 2). Smith (2001) and McGowan & Smith (2008) suggested that this regression had a twofold impact in the marine realm: (*i*) enhanced extinction through contraction of shallow marine ecosystems and increasingly anoxic bottom waters; and (*ii*) decreased preservation of sedimentary rocks, impacting upon on our ability to sample marine assemblages. This regression, combined with increased continental input, and a possible minor ocean anoxic event (Pyenson, Kelley & Parham, 2014), is likely to have been the primary driver behind the apparent faunal turnover and extinction recognised in marine groups.

The latest Tithonian and earliest Cretaceous also experienced several major episodes of large‐scale volcanism and bolide impacts (see Sections (4 and (5, respectively; Fig. 3). Three large bolide impacts are known to have occurred during the Tithonian (Milton *et al.*, 1972; Dypvik *et al.*, 1996; Corner *et al.*, 1997), including one which might have been greater in diameter than the Chicxulub impact at the K/Pg boundary (Misra *et al.*, 2014). Interestingly, no correlation between these impacts and any three‐phase extinction event during the Tithonian has ever been thoroughly investigated (Walliser, 1996; Bambach, 2006), although it was briefly highlighted by Barnes *et al.* (1996) and Upchurch & Mannion (2012). Additionally, there was a large impact in Australia at the Barremian/Aptian boundary (Bron & Gostin, 2012). The Late Jurassic witnessed a series of large volcanic events, with the eruption of one of the single largest volcanoes in our Solar System occurring at the J/K boundary (Sager *et al.*, 2013). This was followed by the emplacement of two large igneous provinces in the Valanginian–Hauterivian (Harry & Sawyer, 1992; Jerram *et al.*, 1999; Seton *et al.*, 2012) and latest Barremian to early Aptian (Renne *et al.*, 1992). Despite both of these existing for longer timescales and being of considerably greater volume than the end‐Cretaceous Deccan volcanism, their potential biotic impacts have never been investigated. With the exception of the Mjølnir impact, these bolide and volcanic episodes were focussed exclusively in Gondwana, or in the Tethys and Panthalassa oceans (Fig. 3). The environmental impacts of these events have received some attention (e.g. Bralower *et al.*, 1994; Wignall, 2001; Weissert & Erba, 2004), although how they relate to the patterns of biotic extinction and diversity we see from the Tithonian–Barremian is less clear. For example, the Paraná‐Etendeka volcanism appears to postdate a Tethyan carbonate platform growth crisis (Föllmi *et al.*, 1994) and calcareous nannoplankton calcification crisis (Erba, 2004), although these events are roughly contemporaneous with the Weissert global carbon cycle perturbation (Erba *et al.*, 2004). Environmental change associated with the Ontong Java Plateau and Aptian OAE1a is, however, linked more closely with a nannoconid crisis and repeated biocalcification crises on Tethyan carbonate platforms (Weissert & Erba, 2004). Following these events, planktonic foraminiferans increased in size and diversity (Premoli Silva & Sliter, 1999).

Atmospheric oxygen and carbon dioxide levels did not change substantially through the J/K boundary (Berner, 2009). However, a rapid cooling of sea surface temperatures is detected in the Tithonian and across the J/K boundary (Weissert & Channell, 1989; Bice *et al.*, 2003; Price & Rogov, 2009; Jenkyns *et al.*, 2012). It is likely that the Cretaceous was warmer (Hay, 2008; Littler *et al.*, 2011; Pouech *et al.*, 2014), suggesting distinct climatic regimes between the Late Jurassic and Early Cretaceous. Increasing sulphur concentrations in the Early Cretaceous marine realm might reflect changes in nutrient input, and are probably unrelated to the late Valanginian–Hauterivian Paraná‐Etendeka volcanism (Callegaro *et al.*, 2014). However, the Otong Java Plateau volcanism is a strong candidate for the increase in sulphur toxicity. Instead, the Etendeka volcanic episode might have been responsible for a positive *δ* ^13^C excursion and higher CO_2_ levels throughout the Valanginian Weissert oceanic anoxic event (Erba *et al.*, 2004), although the environmental impact might have been relatively small compared to other large‐scale igneous events (Dodd *et al.*, 2015). The potential impact that these dramatic events and clear environmental changes might have had on biotic patterns during the Late Jurassic and Early Cretaceous requires future investigation.

### (3) Biotic interactions and evidence for a faunal turnover

#### (a) *Terrestrial realm*

Recent developments in assessing the macroevolutionary and macroecological history of dinosaurs have provided insight into potential selectivity patterns in different subgroups. For example, there is a seemingly selective extinction of larger‐sized dinosaurs (sauropods and theropods) across the J/K boundary (Upchurch *et al.*, 2011; Upchurch & Mannion, 2012; Zanno & Makovicky, 2013; Cobos *et al.*, 2014; De Souza & Santucci, 2014; Carballido *et al.*, 2015). In sauropods, this extinction is focused on broad‐toothed non‐neosauropod eusauropods and narrow‐toothed diplodocids (Barrett & Upchurch, 2005), with just two occurrences known from the Cretaceous (Gallina *et al.*, 2014; McPhee *et al.*, 2016), and is followed by the diversification of rebbachisaurids and titanosauriforms (Upchurch & Mannion, 2012). The earliest Cretaceous therefore represented a ‘transitional’ phase in sauropod evolution (Upchurch *et al.*, 2015b), a pattern also found in Asia, with the replacement of non‐neosauropods by titanosauriforms across the J/K boundary (Wilson & Upchurch, 2009; Mannion *et al.*, 2013).

Evidence of a combined ecological and taxonomic focus of extinction in saurischian dinosaurs, to the exclusion of most ornithischian groups, combined with environmental preferences between different sauropod groups (Mannion & Upchurch, 2010a), suggests that a combination of factors were acting upon dinosaurs at the J/K boundary (Upchurch *et al.*, 2011; Upchurch & Mannion, 2012). These differences between the major herbivorous dinosaur groups potentially relate to different requirements for forage consumption; however, there is little evidence for any major floral perturbations at the J/K boundary (e.g. Barrett, 2014, and references therein), except for a tentative coupling between the decline of cycadophytes and stegosaurs during the earliest Cretaceous (Butler *et al.*, 2009a, 2009b). The only known herbivorous tetanuran theropod lineage in the Jurassic has no known Cretaceous representative (Novas *et al.*, 2015). It has been suggested that medium‐sized theropods underwent a substantial decline across the J/K boundary, and were replaced by larger‐bodied carcharodontosaurids and spinosaurids (Novas *et al.*, 2013; Tortosa *et al.*, 2014). Whereas a literal reading of the fossil record might indicate an Early Cretaceous diversification of smaller‐bodied coelurosaurian theropods (Zanno & Makovicky, 2013; Tortosa *et al.*, 2014; X. Wang *et al.*, 2014*b*), at least a portion of this is undoubtedly an artefact of variation in the degree of Early Cretaceous preservation and the discovery of numerous new species in the Jehol Biota. Furthermore, at least some medium‐sized basal theropod clades persisted into the Early Cretaceous (Sánchez‐Hernández & Benton, 2014), which suggests that part of this extinction selectivity signal might be a product of our poor sampling of earliest Cretaceous terrestrial deposits (Benson *et al.*, 2013; Fig. 4).

This evidence points towards the J/K boundary representing a period of ecophysiologically driven faunal turnover in dinosaurs. Whether or not this was due to competitive displacement or opportunistic replacement, as some groups declined followed by the radiation of new groups, is currently unknown. It is noteworthy that herbivorous groups such as diplodocids and stegosaurs show evidence of a decline, followed by the subsequent diversification of other herbivorous lineages, including ankylosaurs, basal ceratopsians, and iguanodontians, which does not appear to be related to major changes in floral patterns (Butler *et al.*, 2009a, 2009b). This lends support to the opportunistic replacement hypothesis, whereby extinction creates vacant ecospace, which subsequently becomes occupied by newly radiating groups (e.g. Benton, 1996). There is also some evidence for this mode of ecological interaction in mammaliaforms, with multituberculates becoming dominant in the Early Cretaceous, except in localities where similarly herbivorous tritylodontids are present (Averianov *et al.*, 2015).

The apparent Early Cretaceous radiation of diverse groups of avialans, including Enantiornithes and Ornithuromorpha (O'Connor *et al.*, 2011; Wang *et al.*, 2013, 2015; Lee *et al.*, 2014), might have been caused by the release of ecological pressure from the decimation of non‐pterodactyloid faunas at the J/K boundary (Butler *et al.*, 2013), although the timing of these events might be distorted by taphonomic artefacts. Whereas support for this timing comes from evidence of increased diversification rates in pygostylian theropods in the latest Jurassic and earliest Cretaceous (Benson & Choiniere, 2013), combined with sustained decreases in body size (Benson *et al.*, 2014a) and broader occupation of ecological roles (Mitchell & Makovicky, 2014), these diversification studies cannot account for heterogeneous sampling of the fossil record. Additionally, pterosaurs began to occupy increasingly terrestrial environments in the Cretaceous (Butler *et al.*, 2013; Andres *et al.*, 2014), which might represent an ecological reorganisation of flight‐capable faunas at this time. This is supported by evidence for sustained constraint on pterosaur body sizes through the Late Jurassic, potentially through competitive interaction with increasingly diverse avialan faunas (Benson *et al.*, 2014b). The remaining pterosaur lineages after the J/K boundary experienced an increase in morphological disparity, synchronous with that for birds, suggesting a form of competitive interaction to fill ecological morphospace subsequent to boundary extinctions (Butler *et al.*, 2011, 2012, 2013). This ecological expansion is most discernible in groups such as azhdarchoids, which adopted novel aerial morphologies leading to enhanced maneuverability (Frey, Meyer & Tischlinger, 2011).

The diversity dynamics of smaller‐bodied, terrestrial non‐archosaurian tetrapod groups is currently understood less well, but several patterns point to important ecological shifts between the main groups. At the J/K boundary, the majority of rhynchocephalians went extinct, especially those with a piscivorous or molluscivorous diet (Rauhut *et al.*, 2012). Contemporaneous with this extinction is the diversification of several other major lepidosaurian lineages (Marjanović & Laurin, 2013). These clades maintained high ecological plasticity through the J/K boundary, which might have sustained their high diversity compared to more ecologically ‘static’ lineages. Similarly, lissamphibians had acquired a key innovation – neoteny – by the J/K boundary, which might explain their high survivability (Gao & Shubin, 2001). Likewise, mammaliaforms attained several key phenotypic adaptations, particularly regarding sensory organs and dentary specialisations (Heinrich, 1998; Sigogneau‐Russell, Hooker & Ensom, 2001; Kielan‐Jaworowska *et al.*, 2004; Luo, Ruf & Martin, 2012; Wilson *et al.*, 2012; Zhou *et al.*, 2013). A range of significant mammalian lineages, including multituberculates and eutriconodonts, might have competitively or opportunistically replaced more basal forms, including dryolestids and docodonts, at the J/K boundary (Cifelli *et al.*, 2014). It is possible that high ecological diversity provided the basis for the broad survivability of lepidosauromorphs, lissamphibians and mammaliaforms through the J/K boundary, when other larger‐bodied and more specialised terrestrial groups (e.g. dinosaurs) were experiencing a phase of decline. Regional evidence exists for smaller‐scale faunal turnovers, such as that between neosuchian crocodylomorphs and choristoderes in the earliest Cretaceous, which might relate to climatic preferences and/or ecologically selective extinctions (Matsumoto & Evans, 2010; Amiot *et al.*, 2015; Matsumoto, Manabe & Evans, 2015).

For terrestrial invertebrates, much less is known, but a Late Jurassic ‘terrestrial revolution’ in insects, relating to the evolution of phytophagy and parasitism (Labandeira & Currano, 2013), was potentially related to the diversification of new floral groups, as well as an increase in the overall abundance of insects (Labandeira, 2005). Increased origination rates of major Coleoptera groups (e.g. Polyphaga) in the Early Cretaceous support this hypothesis (Smith & Marcot, 2015).

#### (b) *Marine realm*

The low ecological diversity of plesiosaurians and testudines might have been a distinct contributing factor to their decline and turnover at the J/K boundary (Benson *et al.*, 2010; Benson & Druckenmiller, 2014; Rabi *et al.*, 2014), although marine turtles might not have suffered a diversity drop, and non‐marine turtles seem to have increased in diversity (Nicholson *et al.*, 2015). Numerous terrestrial to shallow‐marine basal testudines went extinct across the J/K boundary, including eucryptodirans, plesiochelyids, and eurysternids. This was followed by the subsequent diversification of pancryptodirans and pleurodirans in a geographically structured manner (Hirayama *et al.*, 2000; Cadena *et al.*, 2013; Bardet *et al.*, 2014; Püntener *et al.*, 2014; Nicholson *et al.*, 2015). The radiation of new plesiosaurian lineages immediately after the J/K boundary (i.e. Elasmosauridae and Leptocleididae) is also clear evidence for a within‐group faunal turnover, and conceivably related to the easing of ecological pressure following the gradual extinction of thalattosuchian crocodylomorphs in the Late Jurassic and Early Cretaceous (Young *et al.*, 2010; Mannion *et al.*, 2015). It is likely that this ecological interaction is responsible for the diversification of many major shark lineages around the J/K boundary (Sorensen *et al.*, 2014). Although metriorhynchids exhibited a range of ecologies in the Late Jurassic, their diversity and disparity declined in the earliest Cretaceous (Young *et al.*, 2010). Ecological plasticity might have been important for ichthyopterygians, with high overall ecological diversity of platypterygiines contributing to their persistence through the J/K boundary (Fischer *et al.*, 2013b). The decline in some fully marine reptile groups (i.e. plesiosaurs and thalattosuchians) could also have been driven by a shift in food sources, with a faunal change from holostean to teleostean fish (Steel, 1973; Sallan, 2014), and severe depletion in osteichthyans at the J/K boundary (Friedman & Sallan, 2012).

The J/K boundary further exhibits a dramatic decline in reef communities, illustrated by a distinct turnover from rudist to scleractinian‐dominated systems (Aberhan *et al.*, 2006). This reef decline might also be related to the dramatic rise in shallow to moderately deep infaunal suspension feeders in shallow marine settings (Aberhan *et al.*, 2012). Congruent with this is the dramatic decline of other reef‐dwelling epifauna such as crustaceans (Klompmaker *et al.*, 2013), prior to a faunal turnover in brachyuran decapods that reflects an ecological shift to deeper‐water taxa. However, some reef‐dwelling organisms appear to have been comparatively unaffected (e.g. echinoderms), perhaps facilitated by a high ecological diversity in these groups (e.g. Baumeister & Leinfelder, 1998). In the majority of other marine invertebrate groups, there is less evidence for a faunal turnover, with the J/K boundary instead representing a time of elevated extinction rates, but not accompanied by within‐group ecological reorganisation. These changes, however, are also likely to have been tied to the environmental changes outlined in Section II (b; therefore any attempt to decouple biotic and abiotic factors influencing marine diversity patterns remains problematic.

### (4) Summary

There is strong evidence for a coupled ecological–taxonomic mode of extinction and faunal turnover across the J/K boundary for both small‐ and large‐bodied terrestrial tetrapods. In a range of groups spanning the marine and terrestrial realms, including mammaliaforms, lissamphibians, and ichthyosaurs, ecological specialisation and plasticity plays a clear role, with surviving groups possessing broader morphologies, or key morphological adaptations that appear to be associated with higher survivability rates. For marine tetrapod groups in particular, it appears that the J/K interval represents a staggered cascade model of extinction, with different groups responding in a variety of ways to a range of ecological perturbations, and with fluctuations in sea level possibly acting as the principal driver of change (Hallam & Wignall, 1999). As such, whereas there is evidence for widespread taxonomic replacement and/or faunal turnover in marine groups, this is accompanied by an ecological turnover, with particular lineages being replaced by novel forms capable of adapting to different environments. Whereas very little is known about terrestrial invertebrates during this interval, marine invertebrates also document a period of elevated extinction, focused on shallow‐marine or reef‐dwelling, high‐latitude, sessile taxa. For microfossils, there is some evidence that suggests the spatially structured decline of Foraminifera contributed to the diversification of radiolarian and plankton groups, although the precise mechanism and magnitude of this is currently unknown. The macroevolutionary dynamics of plants during the Late Jurassic–Early Cretaceous remain unclear.

The latest Jurassic to earliest Cretaceous, therefore, represents a relatively poorly understood, but clearly important, period in the history of life on Earth. There is strong evidence for a series of important environmental disturbances through the J/K interval, including dramatic volcanism and bolide impact activity on a scale that rivals the K/Pg mass extinction, coupled with long‐term stresses on Earth system cycles. These stresses pervaded into the Early Cretaceous, and are compounded by additional large‐scale volcanism, bolide impacts, and major shifts in marine environments. The result of this is the almost total reorganisation of marine and terrestrial ecosystems, with substantial evidence for a protracted mode of coupled ecological and faunal turnover. However, the degree to which these environmental and faunal patterns are linked, and therefore supportive of the press‐pulse theory of extinction (Arens & West, 2008), with a combination of gradual changes (‘press’) and sudden, catastrophic disturbances (‘pulse’) altering the composition of ecosystems, remains to be tested.

## V. CONCLUSIONS

1. The Late Jurassic–Early Cretaceous interval represents a time of major biotic upheaval and reorganisation. The precise magnitude of extinction is currently unknown, especially in light of our increasing awareness of the impact of incomplete sampling on the patterns preserved in the fossil record. However, it is clear that the J/K extinction, although severe in multiple groups, was not on the same scale as that for the ‘Big Five’ mass extinctions. What is becoming apparent, though, is that the J/K interval represents a period of elevated extinction, substantially protracted over some 25 million years, and involves the persistent loss of diverse lineages, and the origins of many major groups that survived until the present day (e.g. birds).
2. There is widespread evidence for a major faunal turnover in both the marine and terrestrial realms during the J/K interval. Whereas the effect of this is clearer in larger‐bodied organisms such as dinosaurs, we also see evidence for either competitive displacement or opportunistic replacement in smaller‐bodied groups such as lepidosaurs, lissamphibians and mammaliaforms. There is some evidence that pterosaurs and paravian theropods rapidly diversified and adopted new ecomorphotypes in the Early Cretaceous, including the explosive radiation of the most successful extant tetrapod group, birds, although the precise timing of these events is obscured by varying spatiotemporal sampling of these clades. Low‐latitude and shallow marine to semi‐aquatic faunas, including testudines, crocodylomorphs, and reef‐dwelling and sessile invertebrates, suffered the greatest diversity loss in the marine realm, whereas more mobile taxa with greater dispersal ability, such as ichthyosaurs, appear to have been relatively unaffected.
3. The Late Jurassic–Early Cretaceous was a period of major environmental perturbations that have largely been ignored or overlooked in historical analyses of Mesozoic diversity dynamics, in favour of more ‘exotic’ extinction intervals. A range of evidence indicates the following major changes: (*i*) at least three large bolide impacts in the latest Jurassic, one of which might have been bigger than the end‐Cretaceous Chicxulub impact (Fig. 3); (*ii*) a Late Jurassic–Cretaceous ‘greenhouse’ world, interrupted by a latest Jurassic ‘cold snap’ and corresponding aridity episode; (*iii*) a global drop in sea level to a eustatic lowstand through the J/K boundary (Fig. 2); (*iv*) potentially heightened levels of anoxia, oceanic stagnation, and sulphur toxicity over the J/K boundary; (*v*) a series of repeated ‘biocalcification crises’ in the Early Cretaceous, along with two purported oceanic anoxic events in the Valanginian and Hauterivian; (*vi*) the emplacement of the Paraná and Etendeka (late Valanginian–Hauterivian) and Ontong Java Plateau (Barremian–early Aptian) flood basalts, the latter of which might have been three times as voluminous as the end‐Cretaceous Deccan volcanism; and (*vii*) some of the largest volcanic episodes in the history of the Earth, following the emplacement of the Shatsky Rise supervolcano at the J/K boundary. This series of environmental perturbations warrants further investigation in the context of potential biotic effects throughout this time.
4. The J/K boundary represents an opportunity to investigate the environmental and ecological factors governing recovery (e.g. Wei *et al.*, 2015). Distinct extinction and diversification patterns are clearly recorded in different groups, with a range of potential extrinsic abiotic controls. Additionally, the fact that a faunal turnover at the J/K boundary appears to be coupled with an ecological turnover in many groups, suggests that intrinsic biological parameters, principally regarding acquisition of key ecological characteristics and morphological plasticity and disparity, require further investigation in terms of the effects that these might have had on survivability. For example, low disparity in sauropterygians and testudines is coupled with strong evidence for a faunal turnover, whereas high ecological diversity in ichthyopterygians, lepidosaurs, and mammaliaforms is reflected in high survivability rates across the J/K boundary. This level of complexity necessitates the use of a multivariate approach to assessing macroevolutionary drivers (e.g. Benson & Mannion, 2012).
5. There are major gaps in our current knowledge of biological and Earth systems processes and patterns during the J/K interval. These include the absence of sampling‐standardised diversity trajectories for many terrestrial and marine clades (e.g. plants, terrestrial insects, and small‐bodied tetrapods), and the biotic and abiotic drivers of these patterns. Substantial progress has recently been made in modelling the possible drivers of diversification and extinction, especially in terrestrial tetrapods (Sookias, Benson & Butler, 2012*a*; Sookias, Butler & Benson, 2012*b*; Benson & Druckenmiller, 2014; 2014a, 2014b) and marine invertebrates (e.g. Peters, 2008). Combining these methods with increasingly sophisticated ways of analysing diversity in the fossil record (e.g. Alroy, 2010a, 2014), will provide considerable insight into the macroevolutionary history of life through the J/K boundary.

## Supporting information


**Appendix S1.** List of studies that analyse the magnitude of the J/K boundary extinction for different taxonomic groups. For further details on analytical statistics, see references cited within.
